# Impact of elastic ankle exoskeleton stiffness on neuromechanics and energetics of human walking across multiple speeds

**DOI:** 10.1186/s12984-020-00703-4

**Published:** 2020-06-15

**Authors:** Richard W. Nuckols, Gregory S. Sawicki

**Affiliations:** 1 Joint Department of Biomedical Engineering, UNC Chapel Hill and NC State University, Raleigh, NC USA; 2 John A. Paulson School of Engineering and Applied Sciences, Harvard University, Cambridge, MA 02138 USA; 3 Wyss Institute for Biologically Inspired Engineering, Harvard University, Cambridge, MA 02138 USA; 4 George W. Woodruff School of Mechanical Engineering and School of Biological Sciences, Georgia Institute of Technology, Atlanta, GA 30332 USA

**Keywords:** Locomotion, Wearable robotics, Plantarflexors, Spring-loaded, Lower-limb joints, Biomechanics, Metabolic cost, Compliant

## Abstract

**Background:**

Elastic ankle exoskeletons with intermediate stiffness springs in parallel with the human plantarflexors can reduce the metabolic cost of walking by ~ 7% at 1.25 m s^− 1^. In a move toward ‘real-world’ application, we examined whether the unpowered approach has metabolic benefit across a range of walking speeds, and if so, whether the optimal exoskeleton stiffness was speed dependent. We hypothesized that, for *any* walking speed, there would be an optimal ankle exoskeleton stiffness - not too compliant and not too stiff - that minimizes the user’s metabolic cost. In addition, we expected the optimal stiffness to increase with walking speed.

**Methods:**

Eleven participants walked on a level treadmill at 1.25, 1.50, and 1.75 m s^− 1^ while we used a state-of-the-art exoskeleton emulator to apply bilateral ankle exoskeleton assistance at five controlled rotational stiffnesses (k_exo_ = 0, 50, 100, 150, 250 Nm rad^− 1^). We measured metabolic cost, lower-limb joint mechanics, and EMG of muscles crossing the ankle, knee, and hip.

**Results:**

Metabolic cost was significantly reduced at the lowest exoskeleton stiffness (50 Nm rad^− 1^) for assisted walking at both 1.25 (4.2%; *p* = 0.0162) and 1.75 m s^− 1^ (4.7%; *p* = 0.0045). At these speeds, the metabolically optimal exoskeleton stiffness provided peak assistive torques of ~ 0.20 Nm kg^− 1^ that resulted in reduced biological ankle moment of ~ 12% and reduced soleus muscle activity of ~ 10%. We found no stiffness that could reduce the metabolic cost of walking at 1.5 m s^− 1^. Across all speeds, the non-weighted sum of soleus and tibialis anterior activation rate explained the change in metabolic rate due to exoskeleton assistance (*p* < 0.05; R^2^ > 0.56).

**Conclusions:**

Elastic ankle exoskeletons with low rotational stiffness reduce users’ metabolic cost of walking at slow and fast but not intermediate walking speed. The relationship between the non-weighted sum of soleus and tibialis activation rate and metabolic cost (R^2^ > 0.56) indicates that muscle activation may drive metabolic demand. Future work using simulations and ultrasound imaging will get ‘under the skin’ and examine the interaction between exoskeleton stiffness and plantarflexor muscle dynamics to better inform stiffness selection in human-machine systems.

## Background

The field of wearable robotic devices for improving locomotion performance is quickly expanding. In fact, it has been just over 7 years since a tethered, pneumatically powered ankle exoskeleton was shown to reduce the metabolic cost of walking by 6% below normal, demonstrating for the first time that a wearable robotic device could improve the economy of human locomotion [1]. Since that milestone study that ‘broke the metabolic cost barrier’, the metabolic benefit of exoskeleton assistance has been steadily ticking upward [2]. For example, the benefit of powered ankle assistance was successfully transferred to a portable device that can reduce the metabolic cost of walking by up to 11% compared to normal [3–5]. Ongoing experiments using tethered ankle exoskeletons to tune the timing and magnitude of torque assistance have now shown metabolic benefit up to 12% compared to normal walking [6] and as much as 24% for a unilateral system when compared to zero-torque mode [7]. These experiments suggest that savings of > = 30% for bilateral ankle assistance with optimized torque profiles may be possible for a portable system if the cost of carrying the device and its actuators can be minimized. These ankle-based and powered lower-limb exoskeleton systems targeting other joints [8, 9] accomplish the goal of reducing metabolic cost by transferring net mechanical energy to the user. Alternatively, our research has shown that it is possible to use an unpowered, passive-elastic ankle exoskeleton to reduce the metabolic cost of walking by 7.2% while delivering no net mechanical work [10].

Regardless of differences in the method that lower-limb exoskeleton systems use to deliver mechanical assistance (e.g.*,* passive vs. active; myoelectric vs. impedance control), a common thread across studies has been the limited range of gait conditions examined when evaluating their performance. Most exoskeleton devices have been tested on level ground at one single speed. Although the level-ground, single speed combination has proven an important benchmark used in cross-study comparisons, if the goal is to employ these devices in ‘real-world’ scenarios, then further work in evaluation of exoskeleton assistance under a more comprehensive set of gait conditions is needed. Our world is dynamic - we walk, run, change speeds, and move up and downhill. Thus, it is surprising that only a few exoskeleton devices have been extensively tested under ‘non-baseline’ conditions such as load carriage [4], incline walking [11, 12], and intermediate speed walking [4, 13]. In fact, to our knowledge, no study has explicitly evaluated the effect of walking speed on human-exoskeleton neuromechanics and energetics within a single study. Here, we use a state-of-the-art, tethered, powered exoskeleton system to emulate the dynamics of an unpowered elastic ankle exoskeleton based on our previous work [10], confirm its performance at a single speed, and then extend our evaluation to examine performance across a functionally relevant range of walking speeds. Establishing the limits of passive systems in a rigorous and focused scientific manner is important in and of itself, but will also inform the development of devices that incorporate both passive and active components (i.e.*,* semi-active), which have gained popularity in recent years due to their compact and lightweight form-factor, low power requirements and favorable dynamic response [14–17].

The specific goal of this work was to evaluate how the coupling between walking speed and ankle exoskeleton rotational stiffness influences the user’s neuromechanics and energetics. Our previous research indicates that bilateral elastic ankle exoskeletons that place intermediate stiffness springs in parallel with the human triceps surae and Achilles’ tendon can reduce metabolic cost during level walking at 1.25 m s^− 1^ by up to 7% [10]. Whether this same parallel stiffness is optimal at other walking speeds has yet to be determined. As a starting point for developing hypotheses for how the metabolically optimal ankle exoskeleton stiffness ought to change with walking speed, we refer to the biological system for inspiration.

The ankle is an important contributor to mechanical power output in both walking and running over a range of gait speeds. In normal gait, the muscle tendon unit (MTU) of the ankle joint efficiently contributes approximately half of the positive mechanical power generated by the legs for walking [18]. In late stance, the ankle provides almost the entirety of positive power for redirecting the center of mass [19]. In providing an efficient and powerful ankle plantarflexion moment, the muscle and tendon work in concert as series elements. In early stance, as the MTU lengthens, the Achilles tendon stretches and stores elastic energy against the clutch-like isometric triceps surae muscles [20, 21]. This is followed by a rapid period of muscle contraction and tendon recoil where the tendon provides nearly half the positive power [22]. As such, the ankle joint exhibits mechanics that are similar to the loading and unloading of a torsional spring, especially at slow to medium walking speeds.

The spring-like behavior of the ankle joint can be characterized using the ‘quasi-stiffness’. That is, the combined effect of the stiffness derived from tendons, active and passive elements of muscle, and other connective tissues acting across a joint can be estimated by computing the local slope of the moment-angle curve at any phase of a gait cycle. The literature detailing how biological joint quasi-stiffness changes with gait speed is not entirely clear. In a forward-dynamic walking model, the stiffness of an ankle-foot orthosis (meant to simulate a passive ankle) that minimized metabolic cost of transport was nearly constant over gait speeds of 0.5–0.8 m s^− 1^. However, in this study the simulated walking speeds were much slower than preferred walking speed in able-bodied adults [23]. Experiments on humans with gait speeds of 0.75–2.63 m s^− 1^ suggest that the biological ankle quasi-stiffness increases with increasing walking speed. However, the quasi-stiffness and relative increase in ankle quasi-stiffness with speed depends on the phase of stance in which it is evaluated *(*i.e.*,* early, mid, late stance) [24]. For example, quasi-stiffness in early stance increases from approximately 280 to 500 Nm rad^− 1^ with increased walking speed from 0.75 to 1.9 m s^− 1^, while quasi-stiffness in mid and late stance increases by 250 Nm rad^− 1^ and 40 Nm rad^− 1^ respectively in the same range [24].

Previously, using a portable, unpowered elastic ankle exoskeleton at a single speed (1.25 m s^− 1^) we discovered a ‘sweet spot’ in the relationship between exoskeleton rotational stiffness and the user’s metabolic cost where metabolic rate was decreased by 7.2% with an optimal stiffness of 180 Nm rad^− 1^ [10]. Here, we hypothesized that, for *any* given walking speed, there would be a quadratic, ‘bowl-shape’ relationship between the user’s metabolic rate and exoskeleton stiffness. In other words, at each speed, there exists an optimal exoskeleton rotational stiffness that is not too compliant and not too stiff which minimizes the user’s metabolic rate. Further, in accordance with aforementioned reports indicating that biological ankle quasi-stiffness increases with speed [24], we expected that the metabolically optimal ankle exoskeleton stiffness will also increase with walking speed.

## Methods

### Study participants

Eleven healthy adults (4 female, 7 male; age: 27.7 ± 3.3 years; height: 1.75 ± 0.07 m; mass: 76.8 ± 8.2 kg (mean ± SD)) participated in the study. All participants signed an informed consent to participate in the study which was approved by the Institutional Review Board at University of North Carolina at Chapel Hill.

### Exoskeleton emulator

**Fig. 1:**
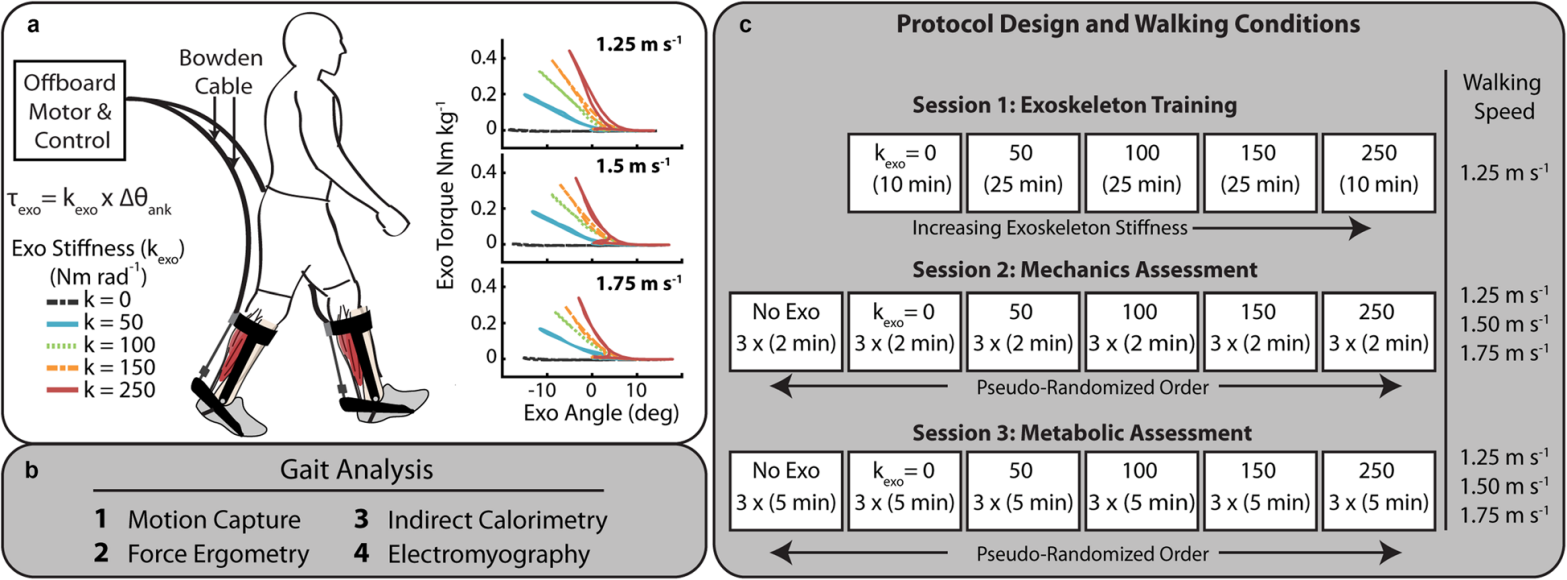
Exoskeleton emulator and study protocol. **(a)** Representative setup of the ankle exoskeleton testing platform for evaluating rotational stiffness plantarflexion assistance. The exoskeleton provided plantarflexion torque to the bilateral ankle end-effectors through off-board motors. The controller emulated elastic rotational stiffness by imposing a torque-angle relationship control law (T_exo_ = k_exo_ * ∆θ_ankle_) where ankle angle was measured with a goniometer. Torque tracking was accomplished by feedback of the applied torque. The ankle exoskeleton work loops for three walking speeds show the change in slope of the work loop representing rotational stiffness **(b)** Biomechanical measurements performed. **(c)** Protocol for the three testing sessions

The exoskeleton emulator was a custom research tool capable of providing bilateral mechanical plantarflexion assistance to the user. A similar device has been demonstrated in previous work [25, 26]. We developed the device in our lab to efficiently and systematically test exoskeleton assistance strategies, and, in this study, we used the system to apply rotational stiffness in parallel to the human plantarflexors via bilateral ankle exoskeletons. The device consisted of three primary components: (1) bilateral ankle exoskeleton end-effectors, (2) benchtop motors and transmission, and (3) control system (Fig. 1).

Each exoskeleton end effector was a lightweight carbon fiber ankle foot orthosis (AFO) that applied plantarflexor torque to the ankle. To allow for flexible testing across participants, we designed the end-effector to be modular such that we could accommodate anthropometric variation in participants’ foot, ankle and shank with four effectors (Short/Small, Short/Large, Tall/Small, Tall/Large). To align the device to the user’s ankle joint center we designed a lockable slider joint to allow for anterior/ posterior positioning of the exoskeleton joint center on the end-effector. The mass of the end-effector without attachment hardware was 415 g.

Two off-board 1.61 kW benchtop motors (Baldor Electric Co, Fort Smith, AR) delivered plantarflexion torque to the bilateral ankle exoskeletons by applying a pulling force to the end of the exoskeleton moment arm through a flexible Bowden-cable transmission. At the end-effector, we terminated the internal cable to an inline tensile load cell (DCE-2500 N, LCM Systems, Newport, UK) and attached to the end effector moment arm (~ 10 cm) through a series elastic element. The motors were positioned behind the participant at approximate shoulder height (1.37 m with respect to treadmill). Through pilot testing evaluation, this height and position provided the best combination of ankle joint range of motion and exoskeleton controllability and limited the interference with camera line of sight.

The exoskeleton controller consisted of a high-level module which calculated a desired torque and low-level module providing torque tracking. The system was implemented through a real-time control package (DS1103 dSpace Inc., Germany) where analog sensor data was sampled at 5 kHz and motor command signals were generated at 500 Hz. A high-level impedance controller determined the desired exoskeleton torque. The impedance controller was designed to emulate a physical passive elastic element capable of providing plantarflexion torque (rotational stiffness) to the ankle akin to our previous portable unpowered elastic ankle exoskeleton [10]. As such, we set the damping coefficient equal to zero, eliminating any dependence of exoskeleton torque on ankle joint angular velocity. We calculated desired torque based off a predefined rotational stiffness and the real-time ankle joint angle:

1 $$ {\tau}_{exo}={k}_{exo}\times \left({\theta}_0-{\theta}_{ankle}\right) $$

where *k*_*exo*_ was rotational stiffness of the exoskeleton (Nm rad^− 1^), *θ*_0_ was the onset angle (rad), and *θ*_*ankle*_ was the real-time ankle joint angle (rad). Similar to the clutch mechanism from [10], the system applied a torque only when the angle was greater than the engagement point (*θ*_0_). A goniometer (5 kHz, 250 Hz Biometrics, Newport, UK) between the foot and shank segment of the exoskeleton measured real-time sagittal plane ankle joint angle, and the onset angle (*θ*_0_) was set to the value recorded at contact of the foot with the treadmill surface. We determined gait state and heel strike by sampling vertical ground reaction force from the instrumented treadmill (Bertec, Columbus, OH). At the detection of toe-off, the cable was pushed out to prevent plantarflexion torque during swing and had a similar effect to the physical pins which disengaged the clutch in the mobile device [10].

The low-level controller was a modified proportional-derivative (PD) controller where torque error was driven to zero using proportional torque error and motor velocity damping feedback in combination with inter-stride learning of torque error [27]. An inline load cell measured exoskeleton force (250 Hz LP Filter) which was converted to a torque via scaling by the exoskeleton moment arm and an optical encoder (E5 Optical Encoder, US Digital, Vancouver, WA) measured motor pulley velocity. An analog velocity command was calculated and sent to the motor controller operating in velocity mode.

### Walking trials

Participants completed testing over three sessions where they walked at three speeds (1.25 m s^− 1^, 1.50 m s^− 1^, 1.75 m s^− 1^) at five ankle exoskeleton rotational stiffness conditions (k_exo_ = 0, 50, 100, 150, 250 Nm rad^− 1^). The low and mid walking speeds were chosen based off commonly used literature values for treadmill walking studies and the knowledge that preferred walking speed of humans usually falls between 1.25 and 1.5 ms^− 1^ [28]. The fast walking speed of 1.75 m s^− 1^ is above preferred walking speed, below the walk to run transition [29], and is a consistent increase from the previous speeds. The order of the three testing sessions was (1) exoskeleton training, (2) gait mechanics, (3) steady-state metabolic energy consumption (Fig. 1c). The imposed waiting period between each testing session was 2–7 days to allow for learning and retention [30].

#### Training

Previous work has demonstrated the importance of training on the acceptance of mechanical assistance in exoskeletons [31–33]. Each participant walked in the exoskeleton at 1.25 m s^− 1^ for a total of 95 min over 5 training trials. The participants walked at each of intermediate stiffness conditions (k_exo_ = 50, 100, 150 Nm rad^− 1^) for 25- min trials and at the lowest and highest stiffness conditions (k_exo_ = 0, 250 Nm rad^− 1^) for 10 min. We collected indirect calorimetry data for each of the trials to monitor changes in metabolic energy use over the course of the training session.

#### Gait mechanics

Participants walked for 2 min at each of the 5 exoskeleton stiffness conditions in a randomized order. We instrumented participants with surface electromyography on the left shank and motion capture markers on the lower limbs and pelvis.

#### Steady state metabolic energy expenditure

We collected indirect calorimetry data for each walking trial. To allow participants’ metabolic rate to reach steady state, each walking trial lasted 5 min. The order of the steady state metabolic conditions was re-randomized.

### Biomechanics measurements

Lower-limb kinematics were measured using a reflective marker based motion capture system (120 Hz, Vicon, Oxford, UK) and participants were instrumented with 44 reflective markers to capture 6-DOF motion (3 rotational, 3 translational) of the foot, shank, thigh, and pelvis. Joint angles were calculated from marker data and joint angular velocities were calculated as the first derivative of the joint angle (Visual 3D, C-Motion, Germantown, MD). Ground reaction forces were captured with a split-belt instrumented treadmill (960 Hz). We performed inverse dynamics analysis to calculate the net joint moments about the ankle, knee, and hip. Analog data was filtered at 25 Hz and marker positions were filtered at 6 Hz. The biological contribution to the total ankle joint moment was calculated by subtracting the measured exoskeleton torque from the total ankle joint moment. Joint angles and moments were reported for the sagittal plane. 6-DOF joint power with a rigid foot was calculated using techniques similar to Zelik et al. [34].

For a given participant, time-domain measurements for each of the 18 conditions were calculated by time normalizing each stride between heel-strike and heel-strike of the subsequent stride. Clean strides (average of 15.5 ± 1.1 strides) from the last 20 s of the walking bout were then averaged together to obtain a single normalized stride for a given condition. Integrated and peak values were calculated prior to intra-stride averaging. Average stride joint moments and powers were calculated by integrating the time-domain moment/power curves and then dividing by stride time. Peak values for a given measurement and condition are the average of the peaks for each stride within that condition. Average data for particular phases of a stride were computed as the time-integral over that phase divided by the time elapsed during that gait phase. To obtain the average moment per unit time (moment rate), we divided again by the stride/stance time over which the integral was taken to obtain the average moment rate (Nm kg^− 1^ s^− 1^). (Note: This rate represents an average moment per unit time and is not a measure of how rapidly the moment is generated).

### Electromyography (EMG) measurements

Muscle activity of the ankle plantarflexors (medial (MG) and lateral (LG) gastrocnemius, soleus (SOL)), ankle dorsiflexors (tibialis anterior (TA)) as well as a hamstrings (biceps femoris long head (BFL)) and quadriceps muscle (rectus femoris (RF)) were measured with surface electromyography (EMG) on the left leg (SX230, Biometrics, Newport, UK). To obtain a linear envelope, the raw EMG data was high-pass filtered at 20 Hz, rectified, and low-pass filtered at 10 Hz.

Integrated EMG (iEMG) was computed as the time-integral of the EMG linear envelope averaged across each stride for a given condition. The amplitude of the EMG envelope and the integrated EMG for each muscle was normalized to the peak amplitude for that muscle observed across all conditions and speeds for each participant. Then, we divided the iEMG by the time period over which it was integrated to get the average EMG. Finally, to get average muscle activation per unit time, we again divided the average EMG by the time period over which it was averaged to obtain average muscle activation (EMG) rate (s^− 1^). (Note: This rate represents an average activation per unit time and is not a measure of how rapidly the muscle turns on and off.)

### Metabolic energy cost measurements

We calculated metabolic power (W kg^− 1^) using a portable indirect calorimetry system (OxyCon Mobile, Vyaire Medical, Mettawa, IL) and applied standard calorimetry equations [35]. To obtain net metabolic power for each condition, we subtracted metabolic power collected during the pretest standing trials from metabolic power for the walking conditions. In each trial, we averaged breath-by-breath data over the last minute of each five-minute trial.

### Rate of average moment and rate of average muscle activation

Inspired by the field of integrative physiology and research findings from Taylor, Kram, and colleagues [36–42], we calculated the ankle moment and muscle activation summary statistics as a ‘rate’ which was the average of the metric per unit time (e.g. average moment per second, or average muscle activation per second). We used this approach because it provides improved understanding of the relationship between metabolic rate and neuromechanical outcomes (e.g., muscle activation or biological joint moment per unit time). Please see Supplementary Text of [43] for in-depth discussion.

### Statistics

Statistical analysis was only performed on exoskeleton conditions in order to isolate the influence of exoskeleton stiffness from the effect of the added mass and other structural features of the device. For joint dynamics, EMG, and net metabolic power, we reported the means and standard error calculated across participants. Net metabolic rate was the primary outcome measure in this study. Based on our previous work [10], we expected a second-order (i.e., quadratic) relationship between net metabolic power (W kg^− 1^) and exoskeleton stiffness (Nm rad^− 1^) for which there would be a minimum at an intermediate stiffness. Therefore, we performed a three-factor, mixed-model ANOVA (random effect: participant; main effect: k_exo_ k^2^_exo_) at each walking speed to test the effect of exoskeleton stiffness on net metabolic power (α = 0.05; JMP Pro, SAS, Cary, NC). For the speeds where we found a significant main effect, we calculated the optimal stiffness as the minimum of the 2nd order regression. We then tested the two stiffness conditions bounding the minimum of the 2nd order regression to determine if there was a significant reduction compared to no assistance (k_exo_ = 0 Nm rad^− 1^) using post-hoc pairwise t-tests with Bonferroni correction. In a secondary analysis, for each walking speed, we performed a two-factor ANOVA (random: participant, main effect: k_exo_) test to examine the relationship between users’ joint biomechanics and muscle activity and ankle exoskeleton stiffness. We performed a number of within-participant, linear, least-squares regression (LLSR) analyses to test for relationships between changes in users’ joint neuromechanics and changes in users’ net metabolic rate with respect to the no assistance (k_exo_ = 0) condition. Finally, in a tertiary analysis, we performed a two-factor ANOVA to test for an effect of walking speed on users’ joint biomechanics and muscle activity (random: participant, main effect: walking speed, k_exo_). A Shapiro-Wilk W test confirmed normality for all tests where relevant.

## Results

### Metabolic cost

**Fig. 2:**
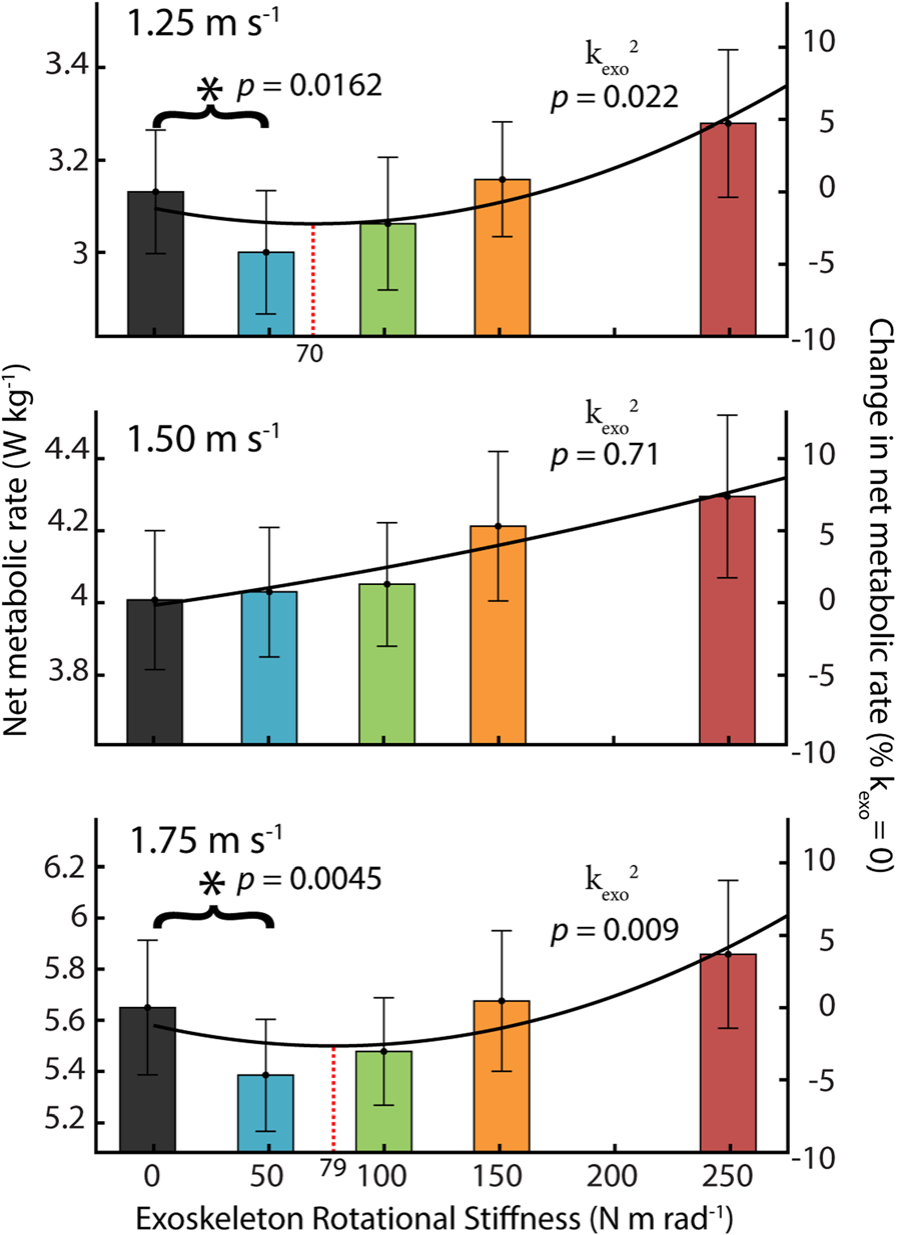
Net metabolic rate across stiffness and speed. Steady state metabolic cost for five exoskeleton stiffness conditions (k_exo_ = 0, 50, 100, 150, 250 Nm rad^− 1^) and three walking speeds (1.25, 1,50, 1.75 m s^− 1^). A significant relationship was found between exoskeleton rotational stiffness and net metabolic rate at slow and fast walking speeds (*n* = 11; mixed model ANOVA with second order term k^2^_exo_; 1.25 m s^− 1^ p^2^_stifffness_ = 0.022;1.75 m s^− 1^ p^2^_stifffness_ = 0.009). No significant relationship was observed for the intermediate 1.50 m s^− 1^ speed. The lowest stiffness (50 Nm rad^− 1^) resulted in metabolic reductions of 4.2 ± 1.7% (mean ± s.e.m) at 1.25 m s^− 1^ and 4.7 ± 1.3% at 1.75 m s^− 1^ (* = paired t-test α = 0.025). The solid line is a quadratic best-fit curve where the stiffness at the minima is indicated by the vertical dashed line

Exoskeleton rotational stiffness reduced the metabolic cost of walking at 1.25 and 1.75 m s^− 1^. At 1.5 m s^− 1^, exoskeleton assistance resulted in increased metabolic cost for all conditions (Fig. 2). A three-factor, mixed-model ANOVA indicated a significant relationship between net metabolic rate and exoskeleton stiffness squared at both 1.25 m s^− 1^ (*n* = 11, k^2^_exo_*p* = 0.022; Net Metabolic Rate = 3.096–0.001 * k_exo_ + 7.047 * 10^− 6^ * k^2^_exo_) and 1.75 m s^− 1^ (k^2^_exo_*p* = 0.009; Net Metabolic Rate = 5.578–0.002 * k_exo_ + 1.318 * 10^− 5^ * k^2^_exo_). We found no significant relationship between exoskeleton stiffness and net metabolic rate for the intermediate walking speed of 1.5 m s^− 1^. (k^2^_exo_, *p* = 0.71). The optimal exoskeleton stiffness, calculated as the minimum of the 2nd order regression model, was 70 Nm rad^− 1^ for slow walking (1.25 m s^− 1^) and 79 Nm rad^− 1^ for fast walking (1.75 m s^− 1^) conditions. We found no optimal exoskeleton stiffness for intermediate speed (1.50 m s^− 1^) walking. For walking speeds of 1.25 m s^− 1^ and 1.75 m s^− 1^, we further analyzed the two stiffness conditions bounding the minimum of the 2nd order regression (50 Nm rad^− 1^ and 100 Nm rad^− 1^ for both speeds) to determine if there was a significant reduction compared to no assistance (k_exo_ = 0 Nm rad). At 1.25 m s^− 1^, we measured a significant reduction in net metabolic rate between the k_exo_ = 0 and 50 Nm rad^− 1^ conditions (− 4.2% CI < -0.4, − 7.8%>; one-tailed paired t-test *p* = 0.0162; α = 0.025) but not 100 Nm rad^− 1^ (− 2.3% CI < 1.6, − 6.2%>; one-tailed paired t-test *p* = 0.108; α = 0.025). At 1.75 m s^− 1^, we measured a significant reduction in net metabolic rate between the k_exo_ = 0 and 50 Nm rad^− 1^ conditions (− 4.7% CI < -1.4, − 7.1%>; one-tailed paired t-test *p* = 0.0045; α = 0.025) but not 100 Nm rad^− 1^ (− 2.6% CI < 1.1, − 6.2%>; one-tailed paired t-test *p* = 0.0551; α = 0.025). Net metabolic power results for individual participants are reported in Supplemental Table 1.

To highlight the effect of exoskeleton stiffness on biomechanics and walking economy, the results focuses primarily on group effects and on the effect of two exoskeleton stiffnesses with respect to the 0 Nm rad^− 1^ (no assistance) condition. The 50 Nm rad^− 1^ condition details what occurs with a stiffness that is low but results in the reduction in metabolic rate for slow and fast walking. The 250 Nm rad^− 1^ condition details what occurs at a perceptually very stiff condition which also resulted in the metabolic maximum.

### Gait neuromechanics at 1.25 m s^− 1^

**Fig. 3:**
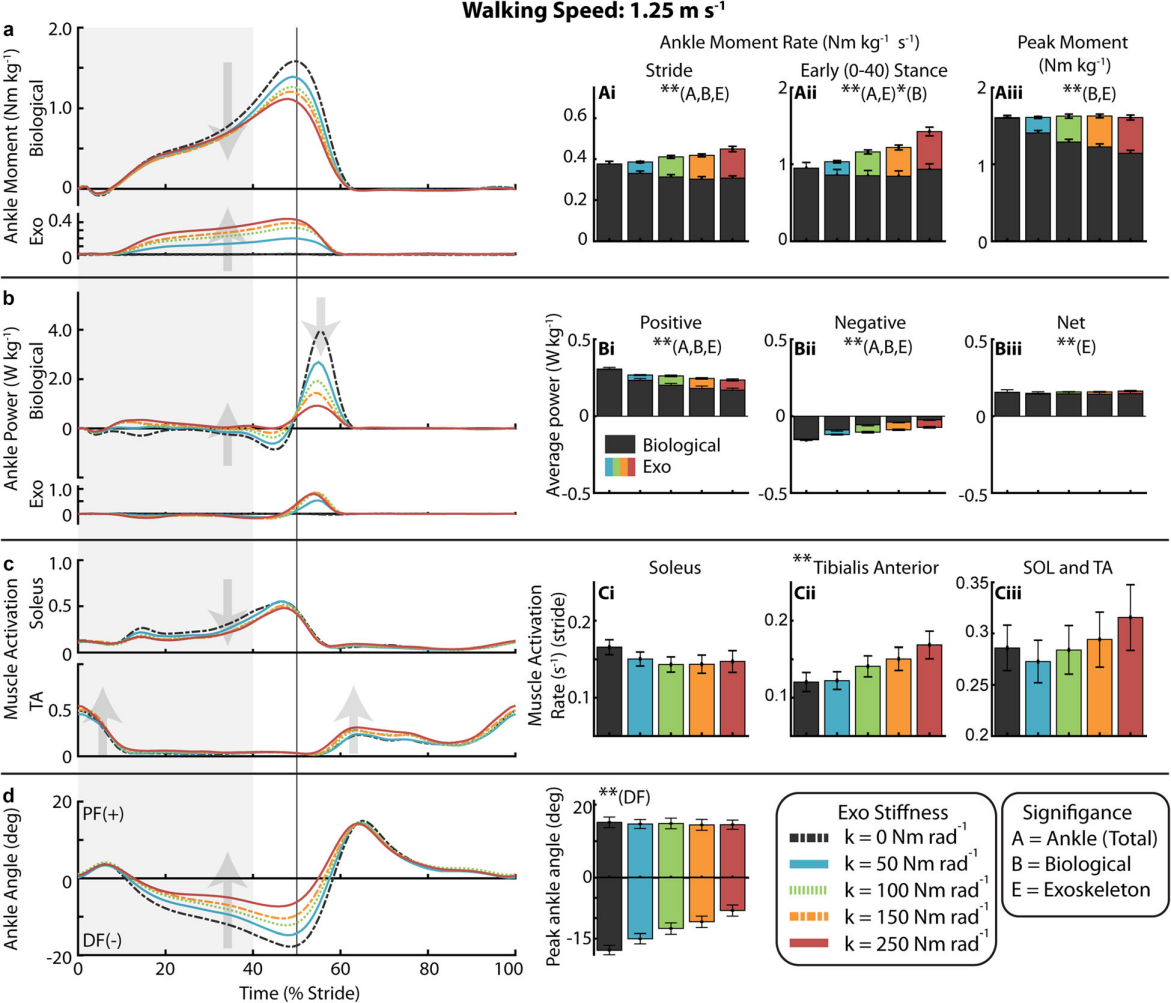
Ankle mechanics and muscle activity during walking at 1.25 m s^− 1^. (**a**) Mass normalized biological moment and exoskeleton torque for each stiffness averaged over participants. Shaded region is early stance (0-40%). Increasing exoskeleton stiffness resulted in increased exoskeleton torque and decreased biological moment. Total ankle moment increased at early stance, but peak moment remained nearly constant. Stacked bar charts represent the average biological (black-lower) and exoskeleton (colors-upper) contribution to total ankle moment rate for gait cycle phases. (**b**) Time series of 6-DOF biological ankle mechanical power and exoskeleton mechanical power for each stiffness. Ankle exoskeleton mechanical power remained consistent at high stiffnesses and biological ankle mechanical power decreased with increased stiffness. Stacked charts represent biological and exoskeleton contribution to total ankle joint mechanical power. Biological ankle net mechanical power was net positive for all conditions and exoskeleton net power was negligible. (**c**) Time-series of soleus and TA muscle activation. TA activation rate increased with stiffness. (**d**) Time series of ankle joint plantarflexion (PF) and dorsiflexion (DF) angle. The magnitude of peak ankle dorsiflexion angle decreased with increased ankle exoskeleton stiffness. [main effect: stiffness ** *p* < 0.0001 *p < 0.05; B = Biological, E = Exo, A = Total Ankle]

With increasing exoskeleton stiffness, the total ankle (exoskeleton torque + biological moment) average moment rate over the stride increased (*p* < 0.0001) as exoskeleton average torque rate over the stride increased (*p* < 0.0001) and despite a decrease in biological ankle average moment rate over the stride (*p* < 0.0001) (Fig. 3a, Ai, Supp. Figure 1). (Note: This rate represents an average moment per unit time and is not a measure of how rapidly the moment is generated). Summary statistics are reported in Supplementary Table 2. Compared to 0 Nm rad^− 1^, 50 Nm rad^− 1^ resulted in a 12% reduction in average biological moment rate over the stride and 250 Nm rad^− 1^ resulted in a 19% reduction. In early stance (0–40%), the total ankle average moment rate increased (*p* < 0.0001) as exoskeleton average torque rate increased (*p* < 0.0001) while biological average moment rate decreased only slightly (*p* = 0.0285). For 250 Nm rad^− 1^ relative to 0 Nm rad^− 1^, the total ankle average moment rate in early stance increased by 52% as the exoskeleton produced 0.49 ± 0.06 Nm kg^− 1^ s^− 1^ (mean ± s.e.m) of torque and biological moment decreased only slightly (Fig. 3Aii). At the time of peak moment, the total ankle joint moment remained constant as exoskeleton torque increased (*p* < 0.0001) and biological moment decreased (*p* < 0.0001) (Fig. 3Aiii). Compared to 0 Nm rad^− 1^, 50 Nm rad^− 1^ resulted in a 12% reduction in peak biological moment and the 250 Nm rad^− 1^ resulted in a 29% reduction.

Average positive mechanical power of the ankle declined with increasing stiffness (*p* < 0.0001), by 30% at 250 Nm rad^− 1^ relative to 0 Nm rad^− 1^ (Fig. 3b, Bi). Negative mechanical power also declined (*p* < 0.0001) (Fig. 3Bii). Exoskeleton average mechanical positive power increased with initial increase in stiffness to 0.036 ± 0.003 W kg^− 1^ at 50 Nm rad^− 1^ but was roughly the same at 0.064 W kg^− 1^ for the 100 Nm rad^− 1^ through 250 Nm rad^− 1^ conditions.

At 1.25 m s^− 1^, SOL activation rate decreased by 9% for 50 Nm rad^− 1^, 13% at 100 Nm rad^− 1^, and 11% at 250 Nm rad^− 1^ compared to 0 Nm rad^− 1^, but the linear effect of stiffness on stride average SOL activation rate was not significant (Fig. 3c Ci). Stride average TA activation rate increased with stiffness (*p* < 0.0001) (Fig. 3Cii). The activation rate of non-weighted sum of stride average SOL and TA (SOL+TA) was reduced by 5% at 50 Nm rad^− 1^ and increased by 10% at 250 Nm rad^− 1^ but the linear effect of stiffness was not significant (Fig. 3Ciii). For LG and MG, we measured an increase in stride average activation rate with increasing stiffness (*p* = 0.0003, *p* = 0.001) which was up 22% at 250 Nm rad^− 1^ relative to 0 Nm rad^− 1^ (Supp. Figure 2B, C). Stride average muscle activation is reported in Supplemental Table 2.

Peak dorsiflexion angle decreased with increasing stiffness (*p* < 0.0001). Angle decreased from 17.8 ± 1.24 degrees at 0 Nm rad^− 1^ to 15.0 ± 1.26 degrees at 50 Nm rad^− 1^ and 8.12 ± 1.39 degrees at 250 Nm rad^− 1^ (Fig. 3d, Di). From 0 to 250 Nm rad^− 1^, stride time decreased (*p* = 0.0063) from 1.09 ± 0.02 s to 1.04 ± 0.04 s, stance time decreased (*p* = 0.0002) from 0.70 ± 0.01 s to 0.66 ± 0.02 s, and the ratio of stance time to stride time (i.e. duty factor) decreased (*p* = 0.0312) from 64.55 ± 0.25% to 63.82 ± 0.26% (Supp. Table 2).

At more proximal joints, increasing exoskeleton stiffness resulted in a shift from knee extension to knee flexion (Supp. Figure 3). Average extension moment rate decreased (*p* < 0.0001) from 0.18 to 0.08 Nm kg^− 1^ s^− 1^ and average flexion moment rate increased from 0.16 to 0.40 Nm kg^− 1^ (*p* < 0.0001) across the range of exoskeleton stiffness (Supp. Figure 3B). Knee average negative and net mechanical power increased as exoskeleton stiffness increased (*p* < 0.0001) (Supp. Figure 3C). Hip flexion (*p* = 0.003) and extension (*p* = 0.0002) moment rate increased, and average positive (*p* = 0.0387) and net (*p* = 0.0447) mechanical power increased with stiffness (Supp. Figure 4B, C). The stance average activation rate of the BFL increased (*p* < 0.0001), by 66% at 250 Nm rad^− 1^, and stride average RF activation rate increased by 28% in the stiffest condition though group effect was not significant (*p* = 0.0646) (Supp. Figure 2E, F).

### Gait neuromechanics at 1.50 m s^− 1^

**Fig. 4:**
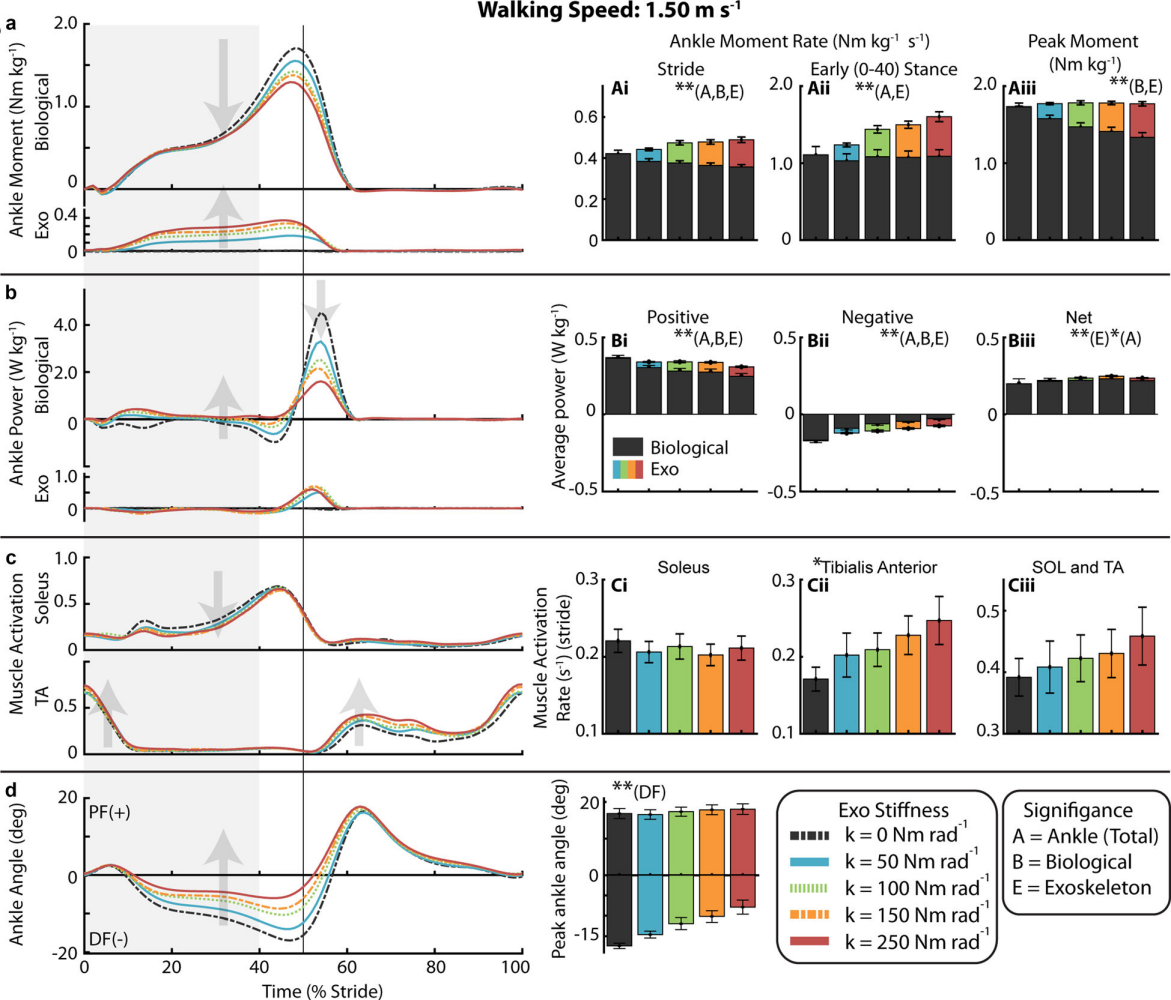
Ankle mechanics and muscle activity during walking at 1.50 m s^− 1^. (**a**) Mass normalized biological moment and exoskeleton torque for each stiffness averaged over participants. Shaded region is early stance (0-40%). Increasing exoskeleton stiffness resulted in increased exoskeleton torque and decreased biological moment. Total ankle moment increased at early stance, but peak moment remained nearly constant. Stacked bar charts represent the average biological (black-lower) and exoskeleton (colors-upper) contribution to total ankle moment rate for gait cycle phases. (**b**) Time series of 6-DOF biological ankle mechanical power and exoskeleton mechanical power for each stiffness. Ankle exoskeleton mechanical power remained consistent at high stiffnesses and biological ankle mechanical power decreased with increased stiffness. Stacked charts represent biological and exoskeleton contribution to total ankle joint mechanical power. Biological ankle net mechanical power was net positive for all conditions and exoskeleton net power was negligible. (**c**) Time-series of soleus and TA muscle activation. TA activation rate increased with stiffness. (**d**) Time series of ankle joint plantarflexion (PF) and dorsiflexion (DF) angle. The magnitude of peak ankle dorsiflexion angle decreasedwith increased ankle exoskeleton stiffness. [main effect: stiffness ** *p* < 0.0001 *p < 0.05; B = Biological, E = Exo, A = Total Ankle]

With increasing exoskeleton stiffness, the total ankle average moment rate over the stride increased (*p* < 0.0001) as exoskeleton average torque rate over the stride increased (*p* < 0.0001) despite a decrease in biological ankle average moment rate over the stride (*p* < 0.0001) (Fig. 4a, Ai). Summary statistics are reported in Supplementary Table 3. Compared to 0 Nm rad^− 1^, 50 Nm rad^− 1^ resulted in a 9% reduction in average biological moment rate over the stride and 250 Nm rad^− 1^ resulted in a 15% reduction. In early stance (0–40%), the total ankle average moment rate increased (*p* < 0.0001) as exoskeleton average torque rate increased (*p* < 0.0001) while biological average moment rate remained constant. For the 250 Nm rad^− 1^ compared to 0 Nm rad^− 1^, the total ankle average moment rate in early stance increased by 46% as the exoskeleton produced 0.52 ± 0.06 Nm kg^− 1^ s^− 1^ of torque and biological moment did not change (Fig. 4Aii). At the time of peak moment, the total ankle joint moment remained constant as exoskeleton torque increased (*p* < 0.0001) and biological moment decreased (*p* < 0.0001) (Fig. 4Aiii). Compared to 0 Nm rad^− 1^, 50 Nm rad^− 1^ resulted in a 9% reduction in peak biological moment and 250 Nm rad^− 1^ resulted in a 23% reduction.

Average positive mechanical power of the ankle declined with increasing stiffness (*p* < 0.0001), by 21% at 250 Nm rad^− 1^ relative to 0 Nm rad^− 1^ (Fig. 4b, Bi). Negative mechanical power also declined (*p* < 0.0001) (Fig. 4Bii). Exoskeleton average mechanical positive power increased with initial increase in stiffness to 0.037 ± 0.004 W kg^− 1^ at 50 Nm rad^− 1^ but was roughly the same at 0.058 W kg^− 1^ for the 100 Nm rad^− 1^ through 250 Nm rad^− 1^ conditions.

Compared to 0 Nm rad^− 1^, SOL activation rate decreased by 7% for 50 Nm rad^− 1^ and by only 4% at 250 Nm rad^− 1^ and the linear effect of stiffness was not significant. (Fig. 4c,Ci). Stride average TA activation rate increased with stiffness (*p* = 0.0032) (Fig. 4Cii). The SOL+TA activation rate increased for all stiffnesses (Fig. 4Ciii) though not significantly (*p* = 0.069). Compared to 0 Nm rad^− 1^, SOL+TA activation rate increased by 4% at 50 Nm rad^− 1^ and 17% at 250 Nm rad^− 1^. For the LG and MG, we measured an average increase in stride average activation rate (*p* < 0.0001) which was up 21% at 250 Nm rad^− 1^ relative to 0 Nm rad^− 1^ (Supp. Figure 2B, C). Stride average muscle activation is reported in Supplemental Table 3.

Peak dorsiflexion angle decreased (*p* < 0.0001) from 17.4 ± 0.7 at 0 Nm rad^− 1^ to 14.6 ± 0.9 degrees at 50 Nm rad^− 1^ and to 7.9 ± 1.7 degrees at 250 Nm rad^− 1^ (Fig. 4d, Di). From 0 to 250 Nm rad^− 1^, stride time decreased (*p* = 0.0010) from 1.00 ± 0.02 s to 0.98 ± 0.02 s, stance time decreased (*p* < 0.0001) from 0.63 ± 0.01 s to 0.61 ± 0.02 s, and the ratio of stance time to stride time decreased (*p* = 0.0008) from 63.1 ± 0.2% to 62.5 ± 0.3% (Supp. Table 3).

At more proximal joints, increasing exoskeleton stiffness resulted in a shift from knee extension to knee flexion for both speeds (*p* < 0.0001) and shift to more negative joint mechanical power (*p* < 0.0001) (Supp. Figure 3). At the hip, there was a slight increase in flexion moment with ankle exoskeleton stiffness (*p* = 0.011) (Supp. Figure 4). The stance average activation rate of the BFL increased (*p* < 0.0001), by 63% at 250 Nm rad^− 1^, and stride average RF activation rate increased by 23% at 250 Nm rad^− 1^ though group effect was not significant (*p* = 0.0594) (Supp. Figure 2E, F).

### Gait neuromechanics at 1.75 m s^− 1^

**Fig. 5:**
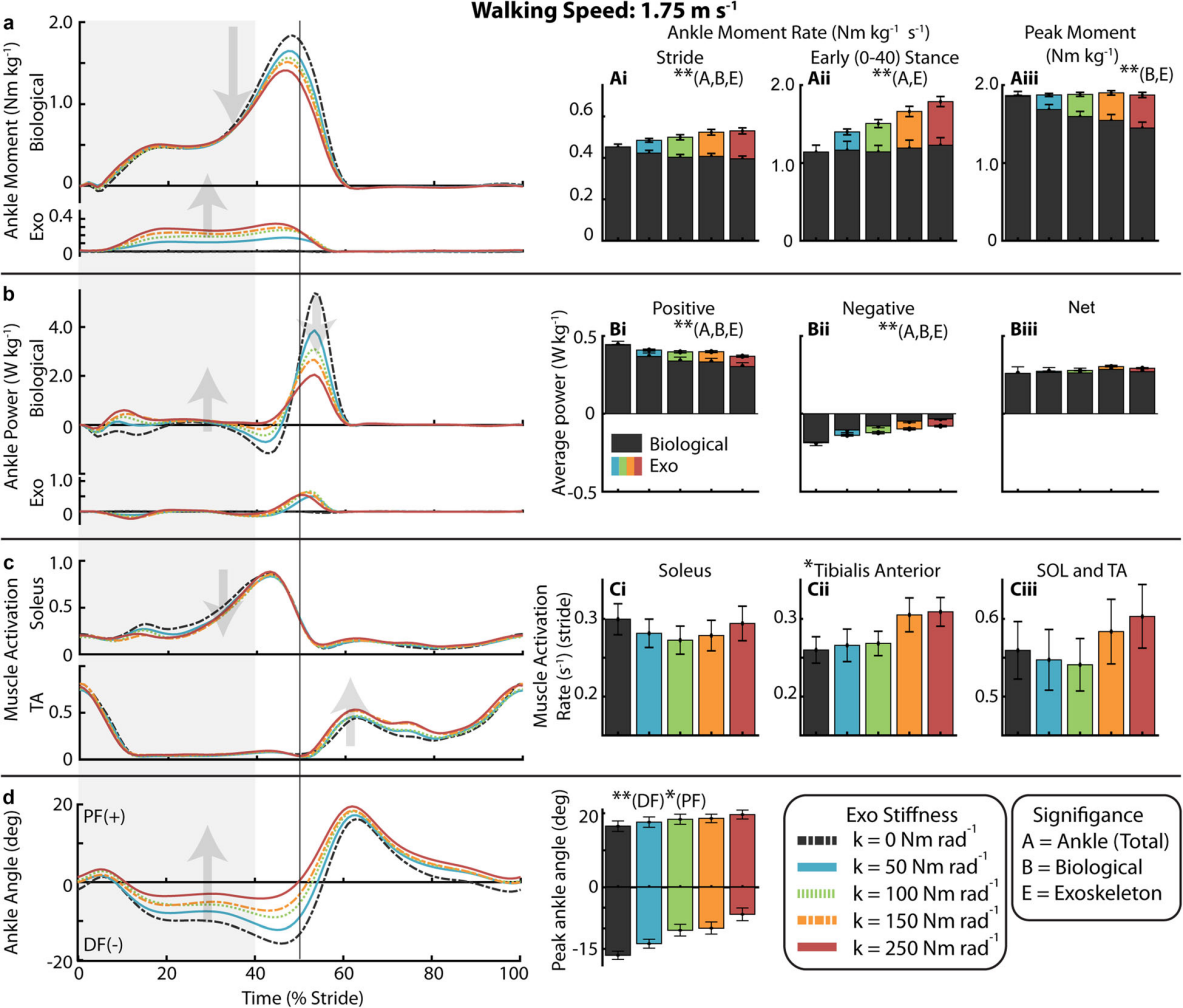
Ankle mechanics and muscle activity during walking at 1.75 m s^− 1^. (**a**) Mass normalized biological moment and exoskeleton torque for each stiffness averaged over participants. Shaded region is early stance (0-40%). Increasing exoskeleton stiffness resulted in increased exoskeleton torque and decreased biological moment. Total ankle moment increased at early stance, but peak moment remained nearly constant. Stacked bar charts represent the average biological (black-lower) and exoskeleton (colors-upper) contribution to total ankle moment rate for gait cycle phases. (**b**) Time series of 6-DOF biological ankle mechanical power and exoskeleton mechanical power for each stiffness. Ankle exoskeleton mechanical power remained consistent at high stiffnesses and biological ankle mechanical power decreased with increased stiffness. Stacked charts represent biological and exoskeleton contribution to total ankle joint mechanical power. Biological ankle net mechanical power was net positive for all conditions and exoskeleton net power was negligible. (**c**) Time-series of soleus and TA muscle activation. TA activation rate increased with stiffness. (**d**) Time series of ankle joint plantarflexion (PF) and dorsiflexion (DF) angle. The magnitude of peak ankle dorsiflexion angle decreased and plantarflexion angle increased with increased ankle exoskeleton stiffness. [main effect: stiffness ** *p* < 0.0001 *p < 0.05; B = Biological, E = Exo, A = Total Ankle]

With increasing exoskeleton stiffness, the total ankle average moment rate over the stride increased (*p* < 0.0001) as exoskeleton average torque rate over the stride increased (*p* < 0.0001) despite a decrease in biological ankle average moment rate over the stride (*p* < 0.0001) (Fig. 5a, Ai). Summary statistics are reported in Supplementary Table 4. Compared to 0 Nm rad^− 1^, 50 Nm rad^− 1^ resulted in a 7% reduction in average biological moment rate over the stride and 250 Nm rad^− 1^ resulted in a 13% reduction. In early stance (0–40%), the total ankle average moment rate increased (*p* < 0.0001) as exoskeleton average torque rate increased (*p* < 0.0001) while biological average moment remained constant. For 250 Nm rad^− 1^ relative to 0 Nm rad^− 1^, the total ankle average moment rate in early stance increased by 58% as the exoskeleton produced 0.56 ± 0.07 Nm kg^− 1^ s^− 1^ of torque and biological moment did not change (Fig. 5aii). At the time of peak moment, the total ankle joint moment remained constant as exoskeleton torque increased (*p* < 0.0001) and biological moment decreased (*p* < 0.0001) (Fig. 5Aiii). Compared to 0 Nm rad^− 1^, 50 Nm rad^− 1^ resulted in a 9% reduction in peak biological moment and the 250 Nm rad^− 1^ resulted in a 22% reduction.

Average positive mechanical power of the ankle declined with increasing stiffness (*p* < 0.0001), by 21% at 250 Nm rad^− 1^ relative to 0 Nm rad^− 1^ (Fig. 5b, Bi). Negative mechanical power also declined (*p* < 0.0001) (Fig. 5Bii). Exoskeleton average mechanical positive power increased with initial increase in stiffness to 0.041 ± 0.007 W kg^− 1^ at 50 Nm rad^− 1^ but was again roughly the same at 0.064 W kg^− 1^ for the 100 Nm rad^− 1^ through 250 Nm rad^− 1^ conditions.

At 1.75 m s^− 1^, the stride average SOL activation rate decreased by 6% for the 50 Nm rad^− 1^, 9% at the 100 Nm rad^− 1^, and 2% at 250 Nm rad^− 1^ compared to 0 Nm rad^− 1^, but the linear effect of stiffness was not significant (Fig. 5c, Ci). Stride average TA activation rate increased with stiffness (*p* < 0.0001) (Fig. 5Cii). The SOL+TA activation rate appeared to be minimized at 100 Nm rad^− 1^ (Fig. 5Ciii) but the linear effect of stiffness on SOL+TA activation was not significant. Compared to 0 Nm rad^− 1^, SOL+TA activation rate was reduced by 2% at the 50 Nm rad^− 1^, 3% at 100 Nm rad^− 1^, and increased by 8% at 250 Nm rad^− 1^. For the lateral (LG) and medial gastrocnemius (MG) muscles, we measured an average increase in stride average activation rate (*p* = 0.0006, *p* < 0.0001) which was up 27% at 250 Nm rad^− 1^ relative to 0 Nm rad^− 1^ (Supp. Figure 2B, C). Stride average muscle activation is reported in Supplemental Table 4.

Peak dorsiflexion angle decreased with increasing stiffness (*p* < 0.0001) from 16.5 ± 1.01 degrees at 0 Nm rad^− 1^ to 13.7 ± 1.1 degrees at 50 Nm rad^− 1^ and 6.61 ± 1.56 degrees at 250 Nm rad^− 1^ (Fig. 5d, Di). Peak plantarflexion angle increased with increasing stiffness (*p* = 0.0116) from 16.5 ± 1.41 degrees at 0 Nm rad^− 1^ to 17.61 ± 1.39 degrees at 50 Nm rad^− 1^ and 19.65 ± 1.21 degrees at 250 Nm rad^− 1^ (Fig. 5Di). From 0 to 250 Nm rad^− 1^, stride time decreased (*p* = 0.0115) from 0.93 ± 0.02 s to 0.91 ± 0.02 s, stance time decreased (*p* = 0.0044) from 0.58 ± 0.01 s to 0.56 ± 0.02 s, and ratio of stance time to stride time did not significantly decrease from 62.3% ± 0.24% (Supp. Table 4).

At the knee, increasing exoskeleton stiffness again resulted in a shift from knee extension to knee flexion (*p* < 0.0001) (Supp. Figure 3B) and shift to more negative joint mechanical power (*p* < 0.0001) (Supp. Figure 3C). The stance average activation rate of the BFL increased (*p* < 0.0001), by 56% at 250 Nm rad^− 1^, and stride average RF activation rate increased by 19% at 250 Nm rad^− 1^ though group effect was not significant (*p* = 0.0715) (Supp. Figure 2E, F).

### Comparison across speeds

The relative contribution of the exoskeleton to total ankle joint mechanics reduced as walking speed increased. With increasing speed, peak total ankle moment (*p* < 0.0001) and peak biological ankle moment increased (*p* < 0.0001) but peak exoskeleton torque decreased (*p* < 0.01) (Supp. Figure 1). From the slowest (1.25 ms^− 1^) to the fastest (1.75 ms^− 1^) speeds, when a stiffness of 250 Nm rad^− 1^ was applied the peak total ankle moment increased by 12% and peak biological ankle moment increased by 26% yet peak exoskeleton torque decreased by 23%. Total positive mechanical power at the ankle increased with speed (*p* < 0.0001) despite little increase in exoskeleton positive mechanical power (0.5%). Only at 50 Nm rad^− 1^ did we measure an increase in exoskeleton positive mechanical power (16%) with increased walking speed.

From slow to fast walking, the ankle dorsiflexion angle decreased (*p* < 0.01) for *all* exoskeleton stiffness conditions, with an average decrease in peak dorsiflexion angle of 25% (Supp. Figure 1). On the other hand, for *all* exoskeleton stiffness conditions, peak ankle plantarflexion angle increased with increasing walking speed (*p* < 0.0001). Stride time decreased (*p* < 0.0001) by 0.15 s (14%) on average from slow to fast speed and stance time decreased (*p* < 0.0001) by 0.12 s (17%) from slow to fast. The ratio of stance time to stride time decreased (*p* < 0.0001) from 64.2 to 61.8% of the gait cycle from slow to fast walking speed (Supp. Table 2–4).

### Association between changes in users’ joint mechanical/muscle activation rates and changes in net metabolic rate

**Fig. 6:**
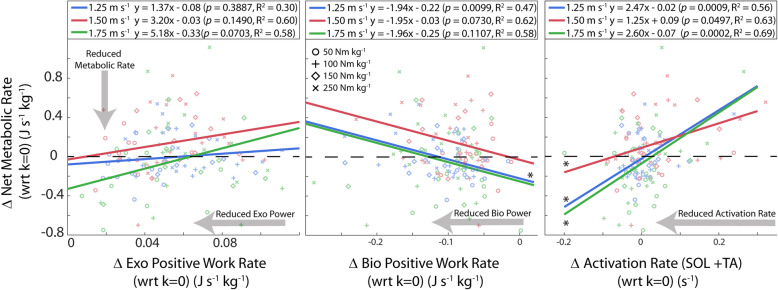
Relationship between lower-limb neuromechanics and whole-body metabolic rate. The relationships between changes in ankle joint and muscle-level neuromechanical rate metrics (x) and the change in net metabolic rate (J s^− 1^ kg^− 1^) (y) during walking with exoskeletons over a range of walking speeds and stiffness values, k_exo_. For each metric, Δ represents the difference from the k_exo_ = 0 condition (i.e.*,* the effect of increasing exoskeleton stiffness) with positive values indicating an increase. We used a within-participant linear regression analysis to determine whether relationships were significant (*p < 0.05), and we report the line of best fit to the data and the R^2^ value of the fit. Statistics on the lines of best fit are also reported in Table 1. Note that work rate (J s^− 1^) and power (W) are synonymous

Changes in users’ SOL+TA muscle activation rate due to exoskeleton assistance were significantly correlated with measured changes in users’ net metabolic rate (Fig. 6). This relationship held for the slow (1.25 m s^− 1^: *p* = 0.0009, R^2^ = 0.56), intermediate (1.50 m s^-1:^*p* = 0.0497, R^2^ = 0.64), and fast walking speed (1.75 m s^-1:^*p* = 0.0002, R^2^ = 0.69). Changes in SOL+TA activation rate were more highly correlated with changes in net metabolic rate when compared with measured changes in biological moment rate, biological power (i.e.*,* mechanical work rate), or exoskeleton power (Fig. 6 and Table 1).

**Table 1:** Relationship between lower-limb joint and muscle-level neuromechanics and whole-body metabolic rate

Gait Cycle Phase	1.25 m s^− 1^	1.50 m s^− 1^	1.75 m s^− 1^
Δ Exoskeleton Positive Work Rate (J kg ^− 1^ s^− 1^)	*p* = 0.3887	*p* = 0.1490	*p* = 0.0703
Δ Biological Positive Work Rate (J kg ^− 1^ s^− 1^)	***p*** **= 0.0099, R**^**2**^ **= 0.47**	*p* = 0.0730	*p* = 0.1107
Δ Biological Moment Rate (N kg ^− 1^ s^− 1^)	*p* = 0.6574	*p* = 0.0502	*p* = 0.1273
Δ Soleus + Tibialis Anterior Activation Rate (unitless s^− 1^)	**p = 0.0009, R**^**2**^ **= 0.56**	**p = 0.0497, R**^**2**^ **= 0.63**	**p = 0.0002, R**^**2**^ **= 0.69**

The relationships between changes in ankle joint and muscle-level neuromechanical rate metrics (x) and the change in net metabolic rate (W kg^− 1^) (y) during walking with exoskeletons over a range of walking speeds and stiffness values, k_exo_. For each metric, Δ represents the difference from the k_exo_ = 0 condition (i.e.*,* the effect of increasing exoskeleton stiffness) with positive values indicating an increase. We used a within-participant linear regression analysis to determine whether relationships were significant (*p < 0.05), and we report the line of best fit to the data and the R^2^ value of the fit. Data showing actual lines of best fit can be found in Fig. 6. Note that work rate (J s^− 1^) and power (W) are synonymous

### Training

Results from the training day indicate that participants adapted in a metabolically beneficial manner only in the 50 Nm rad^− 1^ exoskeleton stiffness condition (Supp. Figure 5). Over the training period, for 50 Nm rad^− 1^ participants on average reduced their net metabolic power by 6.7% (*p* = 0.017, two-tailed paired t-test). Two testing sessions later, the net metabolic rate during the steady-state testing day was on average 2.9% lower than the rate at the end of the training day though not significantly so (*p* = 0.6, two-tailed paired t-test). Conversely, we measured an increase of 1.7% in net metabolic rate over the testing period for the three stiffest exoskeleton conditions. Values recorded during the steady-state metabolic data collection sessions were, on average, 7.8% lower for the three high stiffness conditions when compared to the training day.

## Discussion

Our data suggest that passive elastic ankle exoskeletons can reduce metabolic cost at multiple, but not all, walking speeds (Fig. 2). The lowest tested exoskeleton rotational stiffness condition (50 Nm rad^− 1^) resulted in a metabolic reduction of − 4.2% at slow (1.25 m s^− 1^) and − 4.7% at fast (1.75 m s^− 1^) walking speeds when compared to no assistance (i.e. k_exo_ = 0 Nm rad^− 1^ = zero-torque). The results from this study for walking at 1.25 m s^− 1^ support findings from our previous study where rotational stiffness was applied using physical springs rather than the emulated elastic system we present here [10]. At slow and fast walking speeds, we confirm a second order relationship (k^2^_exo_) between exoskeleton rotational stiffness and the users’ net metabolic power, indicating a ‘sweet-spot’ such that balancing a trade-off between very high stiffness (not too stiff) and very low stiffness (not too compliant) is necessary to achieve metabolic benefit. Surprisingly, no exoskeleton stiffness condition resulted in an average decrease in metabolic cost at the intermediate speed (1.5 m s^− 1^). Rather, for walking at 1.5 m s^− 1^, we observed a monotonic linear increase in users’ net metabolic rate with increasing exoskeleton stiffness.

Using our extensive set of human neuromechanical data across a range of functionally relevant walking speeds, we endeavored to gain a comprehensive understanding of the effect of passive-elastic ankle exoskeleton assistance on lower-limb joint mechanics and muscle activity and establish which factors drive changes in users’ whole body metabolic rate. Traditionally, exoskeleton effects have been analyzed at a single speed where it is assumed that even with the addition of exoskeleton assistance, the stride time remains relatively consistent. The traditional approach calculates an average moment per stride (or period of interest) which is then compared to the metabolic rate (energy per second). For conditions with constant stride times, this approach may be sound. However, to enable the comparison of metabolic rate and other neuromechanical metrics across a wide range of exoskeleton stiffness and multiple speeds, the assumption of constant stride time is no longer sound, and the comparison of average moment per stride (no indication of time) to energy per time did not seem appropriate. Inspired by the field of integrative physiology and research findings from Taylor, Kram, and colleagues [36–42], we calculated the rate of the metric of interest (e.g. ankle moment, muscle activation) computed as the average of the metric per unit time (e.g. average moment per second, or average muscle activation per second). Upon inspection, the changes in rate metrics that were observed across exoskeleton stiffness and walking speed conditions were likely due to both changes in the average magnitude of the metrics and the duration of the stride (Supp. Tables 2–4). We used the rate of the metric of interest (average per second) for a more apt comparison to metabolic rate (J s^− 1^) (See [43] for a similar approach).

With this approach, we sought to determine which factors drive changes in users’ whole body metabolic rate across speeds. Whole body metabolic rate during walking can in part be attributed to the energetic cost of active muscle contraction (i.e., use of ATP for cycling cross-bridges) across the lower limbs. Recent work in muscle energetics shows that muscle stretch-shorten cycles requires similar amounts of energy to an isometric contraction over the same duration which suggests that muscle energy use may be more closely tied to costs of force rather than work production [44]. In an exoskeleton hopping study, metabolic cost was reduced when muscle force was reduced but muscle work remained constant [45]. Thus, we anticipated that much of the metabolic improvement from an elastic ankle exoskeleton would be provided by reducing biological ankle joint moment and therefore plantarflexor muscle-tendon force and ultimately active muscle volume of the calf muscles [46]. This would suggest that applying more torque assistance to reducing biological muscle loading as much as possible should be the best way to maximize metabolic benefit of an exoskeleton.

Our results suggest that to some extent the simple solution that exoskeletons reduce biological joint moments, muscle-tendon forces and active muscle volume appears correct. Indeed, the conditions where we achieved a reduction in metabolic cost (k_exo_ = 50 Nm rad^− 1^ at 1.25 and 1.75 m s^− 1^) also exhibited measured reductions in biological ankle moment (Figs. 3a and 5a, Supp. Figure 1B) and reduced soleus muscle activity (Figs. 3c and 5c, Supp. Figure 2A). However, our data also suggest that across walking speeds, more assistance (i.e.*,* higher exoskeleton stiffness) is not better, and that when a ‘sweet-spot’ where metabolic benefit is achieved is found, it results from balancing appropriate amounts of exoskeleton torque assistance with deleterious side effects that can manifest via altered motor coordination, limb-joint kinetics and muscle-tendon dynamics [47–50]. For example, when further evaluating users’ physiological response to elastic ankle exoskeletons at each speed in isolation, qualitative trends in the effect of increasing device stiffness on ‘non-local’ neuromechanics (i.e.*,* not ankle plantarflexors) were consistent and pointed toward potential metabolic penalties. Increasing exoskeleton assistance consistently resulted in increased MG, LG, and TA muscle activity (Supp. Figure 2B, C, D). In addition, at the knee, for each of the speeds we studied, we measured a substantial increase in muscle activation from the BFL (Supp. Figure 2E) that was accompanied by an expected concomitant shift from knee extension to knee flexion moment (Supp. Figure 3B). Despite these consistent qualitative, if not quantitative, trends in users’ joint-level and muscle-level neuromechanical response to increasing exoskeleton stiffness, it was difficult to find a simple mechanical explanation for the differences in the observed relationships between exoskeleton stiffness and users’ net metabolic rate at both slow and fast speeds (‘bowl-shaped’ at 1.25 and 1.75 m s^− 1^) versus at the intermediate walking speed (monotonically increasing at 1.5 m s^− 1^).

One possibility was that the ankle exoskeleton mechanical performance across stiffness could have been speed dependent, rendering it least effective at 1.5 m s^− 1^. In fact, contrary to the dynamics of the biological ankle, where both the moment and power increase steadily with speed [18, 51], exoskeleton torque actually *decreased* with increasing walking speed (Supp. Figure 1B). These results are perhaps not surprising considering passive-elastic exoskeleton torque is driven by the user’s ankle joint kinematics and peak ankle dorsiflexion is known to decreases with increasing walking speed [52]. Indeed, our results indicate that peak ankle dorsiflexion decreased by 11% in unassisted conditions across speed and by as much as 43% in the stiffest exoskeleton within a given speed. As a result, we observed very little increase in exoskeleton positive/negative power with speed and increases in exoskeleton power were small compared to increases in biological power calculated from our unassisted trials (47%) and previous work performed over the same speed range (45%) [18].

Despite the inability of our passive-elastic ankle device to increase its mechanical output with increasing speed, we found no relationship between exoskeleton positive power (= positive work rate) and users’ net metabolic rate (Fig. 6, Table 1). This is in contrast to previous results from active devices, where exoskeleton mechanical power output seems to drive user benefit [4, 5, 7, 26, 53]. Perhaps this incongruity stems from fundamental differences in the timing of assistance torque, as most active ankle systems are specifically designed to inject positive power to during late stance push-off. Our passive-elastic ankle exoskeletons apply torque to offload biological plantarflexor force with onset much earlier in stance. However, neither changes in users’ biological positive power (i.e. work rate) or biological moment rate could explain the changes in net metabolic rate across all speeds (Fig. 6 and Table 1). The lack of relationship between changes in users’ biological moment rate and metabolic rate may seem to contradict the idea that muscle force drives metabolic energy consumption; however, ankle moment is calculated as an externally measured net moment and does not account for co-contraction of antagonists muscles crossing the joint. Thus, an increase in co-contraction of dorsiflexors (e.g.*,* TA) would result in underestimation of plantarflexor muscle force when derived from biological ankle moment. Indeed, we measured a significant increase in TA muscle activity with increasing exoskeleton stiffness which suggests an increase in co-contraction across the ankle. Therefore, in this study, changes in biological ankle moment were likely a poor representation of changes in plantarflexor muscle force and may help explain the poor relationship between changes in biological moment and changes in net metabolic rate.

A closer look at changes in ankle muscle activity reveals a nuanced trade-off between beneficial reductions in plantarflexor activity and costly increases in antagonist dorsiflexor activity across the range of exoskeleton stiffnesses we tested (Figs. 3c, 4c, and 5c). In fact, we find that the change in SOL+TA activation rate universally explains the bowl-shape net metabolic rate versus exoskeleton stiffness relationship at 1.25 m s^− 1^ (R^2^ = 0.56) and 1.75 m s^− 1^ (R^2^ = 0.69) and also the lack of bowl-shape relationship at 1.5 m s^− 1^ (R^2^ = 0.64) (Fig. 6 and Table 1). Although there was no statistical linear effect of stiffness on SOL+TA activation, the intermediate walking speed (1.50 ms^− 1^) was the only condition in which the data suggest that SOL+ TA activation was not reduced. While the data suggest that SOL activation rate decreased at 50 Nm rad^− 1^ for all three speeds, TA activation rate was substantially increased at the intermediate speed (18%) in comparison to the slight increase (~2%) in TA activation rate at slow (1.25 ms^− 1^) and fast (1.75 ms^− 1^) walking speeds. The changes in activation rate that were observed across exoskeleton stiffness and walking speeds were likely due to both changes in the average magnitude of the activation and the duration of the stride (Supp. Tables 2–4).

As mentioned earlier, and consistent with our previous experimental [10] and modeling/simulation [47] results for walking at 1.25 m s^− 1^, our data here show that humans respond to increasing ankle exoskeleton stiffness with deleterious side effects manifesting via altered motor coordination (Supp. Figure 2), limb kinetics (Supp. Figs, 1, 3, and 4), and muscle-tendon dynamics across the entire range of normal walking speeds (1.25–1.75 m s^− 1^). But why is it that ‘more is not better’? Or more explicitly, what physiological mechanisms may be preventing participants from converting larger and larger local reductions in biological plantarflexor muscle loading to metabolic savings? First, we note a trend toward diminishing returns. Although the exoskeleton stiffness increased by 67% between the k_exo_ = 150 and 250 Nm rad^− 1^ conditions the peak exoskeleton torque increased by only 13% (Fig. 3a). Furthermore, we observe that participants compensated by decreasing the amount of ankle dorsiflexion (Supp. Figure 1), but the mechanism for this compensation is unclear. One potential explanation is that the user is unable to ‘turn down’ their muscle activation in proportion to the level of assistance provided. Joints (and limbs) tend to maintain constant stiffness during a given locomotion condition [54]. This is potentially accomplished through a combination of physiological sensors including length (spindle organs) and force (Golgi tendon organs) sensitive transducers that are responsible for maintaining a nearly constant ratio between changes in muscle force and length [54, 55]. In our data, ankle moment decreased from which we can predict a decrease in plantarflexor muscle force. On the other hand, studies suggest that muscle fascicle lengths become longer with increasing exoskeleton assistance [43, 47]. Thus, a conflict or mismatch in sensory feedback via Golgi tendon and muscle spindle organs may constrain motor adaptation in response to assistive forces. Much of walking is automatic and unconsciously controlled though spinal level central pattern generators, reflexes, cerebellar regulation, and the brainstem [56], and users of the exoskeleton may not be able to turn off their plantarflexors as would be required to obtain maximal benefit or coordination with the exoskeleton. Further work in humans and animals into the role of feedforward/feedback mechanisms and how they are modulated during perturbed walking would be insightful and help understand constraints on motor adaptation during walking with exoskeletons.

Another potential side effect of applying exoskeleton assistance was that the normally efficient muscle-tendon dynamics could become ‘detuned’. Muscles dynamics are governed by intrinsic muscle properties where force production and economy are dependent upon the length of a muscle and its contraction velocity [57, 58]. When exoskeleton assistance is applied, biological moment and thus muscle-tendon force decreases, while strain on the tendon decreases. Modeling and experimental studies of hopping [45, 48, 59] and walking [43, 47, 50] with ankle exoskeletons suggest that muscle lengths are in fact longer and undergo increased excursion with increased exoskeleton assistance, and this could limit muscle force capacity and metabolic economy. Additionally, the biological system may be resistant to increased muscle strain to avoid injury and compensations may arise to limit range of motion [60–62]. A closer look at the effect of elastic ankle assistance on plantarflexor muscle fascicle dynamics may give additional insights into how external forces applied in parallel with biological muscle-tendon units could be controlled to steer individual muscle dynamics [43, 46].

At the joint level, in early stance, the application of elastic exoskeleton torque did not significantly reduce the biological moment (Figs. 3aii, 4aii, and 5aii), but rather enhanced the amount of total ankle moment (i.e.*,* augmentation) (Supp. Figure 1B). In late stance, on the other hand, the exoskeleton reduced the biological moment while the peak total ankle moment remained fairly constant (i.e.*,* replacement) (Figs. 3aiii, 4aiii, and 5aiii). These findings raise the question if, as literature suggests, humans act to maintain a constant total ankle joint moment during walking [63] then why was the total ankle moment increased during the beginning of stance? One possible explanation is that the biarticular MG and LG muscles become ‘overactive’ to prevent hyperextension of the knee (Supp. Figure 2 B, C). Due to the kinetic chain within the lower limbs, the plantarflexion torque of the exoskeleton also generates an extension moment at the knee through dynamic coupling [64]. Perhaps as a compensation for the increasing knee extension moment with increasing exoskeleton stiffness, MG and LG increased activity that may have served as a countermeasure to push the knee towards flexion (Supp. Figure 2B, C; Supp. Figure 3B). The increased activation of the BFL, which is a knee flexor, also supports the idea that additional muscle activation was required for knee flexion (Supp. Figure 2E). The effect exists for the entire stride but may be more critical in early stance when the ground reaction force vector briefly passes in front of the knee. The gastrocnemius moment arm is also larger at the knee for more extended postures [65], giving it more leverage during early stance. It may be possible to avoid costly compensations by using biarticular exoskeleton designs that incorporate a knee component designed to act in a similar manner to the gastrocnemius and compensate for additional load on knee flexors derived from assistive ankle torques [66]. Alternatively, an exoskeleton with a non-linear or piecewise linear stiffness profile where ankle stiffness stayed low until the ground reaction force vector passed behind the knee could also be a potential solution. Unfortunately, delaying the onset angle for the exoskeleton spring or limiting stiffness also has the effect of limiting the amount of energy that can be stored and returned in the device.

Contrary to our expectation, we did not find that optimal exoskeleton stiffness increased substantially with walking speed. Based on the computed exoskeleton stiffness at minimum net metabolic rate from our fitted regressions, we estimate that the optimal stiffness for slow (70 Nm rad^− 1^ at 1.25 m s^− 1^) and fast (79 Nm rad^− 1^ at 1.75 m s^− 1^) walking speeds were similar. The increase in optimal exoskeleton stiffness that we measured (13%) was not proportional to the estimated speed-dependent increase in ankle quasi-stiffness during plantarflexion (21%) or dual-flexion (i.e., late dorsiflexion) (46%) calculated from regression models derived from human walking data [24]. Instead, the 13% increase in optimal exoskeleton stiffness was in closer agreement with the 10% increase in ankle quasi-stiffness during the dorsiflexion phase [24]. This discrepancy can likely be explained by the role of muscle and tendon during stance. During late dorsi-flexion (dual-flexion) and plantarflexion, the contraction of the soleus muscle likely modulates the quasi-stiffness of the joint while the person adapts to walking speed. As walking speed increases, muscle activity increases, plantarflexion begins earlier, and quasi-stiffness in late stance is higher. In early stance during dorsiflexion, the soleus muscle produces force isometrically [20] and allows the Achilles tendon to stretch against it to store energy [22]. Thus, the Achilles tendon (and not concentric muscle contraction) is likely responsible for a large percentage of ankle joint rotational stiffness in early stance, and stiffness during this period is less likely to be actively modulated with speed. Similar to a biological tendon, our passive exoskeleton has no ‘muscle’ and is not capable of modulating stiffness in late stance to adapt to increased walking speeds. Therefore, in hindsight, it may not be that surprising that the optimal exoskeleton stiffness tracks speed-dependent changes in ankle quasi-stiffness during early stance dorsiflexion, when the biological tendon stiffness dominates the quasi-stiffness behavior of the ankle. A positive consequence of the relative invariance in optimal exoskeleton stiffness at slow and fast walking speeds is that even though low-powered clutch-spring systems are being developed that can switch stiffness step by step [67], designing for variable speed conditions becomes less challenging and may not even be necessary.

The changes in SOL+TA activation explain why metabolic reduction was not obtained at intermediate walking speed (1.5 m s^− 1^); however, the mechanism behind the difference in muscle response at intermediate speed versus the other speeds is less clear. Changes in joint mechanics were similar for all three speeds with differences observed in terms of timing or amplitude that were merely exacerbated at the highest walking speed. This suggests to us that perhaps the differential effect of exoskeleton stiffness on users’ net metabolic rate at 1.5 m s^− 1^ was due to muscle-tendon dynamics that were not evident in joint-level data. Previous literature suggests that preferred walking speed in adults is close to 1.42 m s^− 1^, and this speed aligns closely with that which minimizes the metabolic cost of transport [28]. Humans also select step frequencies that reduce energetic cost of walking [68, 69]. The structure of the ankle is important for maximizing muscle efficiency [70, 71] (i.e.*,* minimizing metabolic cost) and models of walking have suggested that walking efficiency is maximized when step length and frequency are matched to target ankle stiffness [72]. Therefore, it is possible that, nominally, ankle plantarflexors are preferentially ‘tuned’ to participants’ preferred walking speed. In this study, preferred walking speed collected from participants using a 10 m over ground test was 1.39 ± 0.04 m s^− 1^ and the average metabolic cost of transport with no exoskeleton assistance at 1.25, 1.50, and 1.75 m s^− 1^ was 2.5 ± 0.11, 2.67 ± 0.13, and 3.22 ± 0.15 J m^− 1^ respectively. Thus, we suspect that the muscle-level ‘detuning’ associated with the exoskeleton assistance may have been more pronounced at the 1.5 m s^− 1^ walking speed due to its proximity to the preferred (and perhaps most economical) speed.

One difference between these study results and our previous study on passive elastic ankle exoskeletons was that the optimal stiffness reported in this study (50 Nm rad^− 1^) is substantially lower than the reported optimal stiffness from the prior work (180 Nm rad^− 1^) [10] . However, the stiffness values previously reported reflected the stiffness of exoskeleton spring elements rather than the stiffness of the whole exoskeleton system. Conversely, the exoskeleton testbed we used here (Fig. 1) imposes a desired torque/angle relationship rather than relying on physical components to provide stiffness making it less sensitive to structural stiffness and deformation of the exoskeleton frame. When deformation in the previous system is accounted for by measuring stiffness from the exoskeleton’s torque/angle relationship, the optimal stiffness for 1.25 m s^− 1^ walking speed was reduced to ~ 80 Nm rad. This is close to the optimal value we found here based on the stiffness at minimum net metabolic power from the regression at the same speed (69 Nm rad^− 1^). We note that there may still have been some movement between the human and device that was not accounted for in our system, but we believe that stiffness values reported here give a more accurate portrayal of the applied rotational stiffness than had been previously reported.

We acknowledge that our study has limitations. In terms of the hardware, our exoskeleton emulator behaved more like an ideal spring rather than a physical spring or a biological spring, which would be expected to dissipate energy in each cycle. Achilles tendon hysteresis, for example, could be as much as 15% per cycle [73], though the true value may be smaller and is still debated [74]. Thus, although our ‘exo-tendon’ did not emulate the exact energy cycle of a biological tendon, our intention was to tightly control stiffness, and vary it systematically, so that we could measure its effect on users’ physiological response. Small amounts of work were generated/dissipated, but we estimate the impact of these amounts of mechanical energy were less than 0.1% of the total net metabolic power. Future work could consider damping in addition to stiffness in the exoskeleton impedance control law to regulate the amount of energy dissipated by the exoskeleton.

Another limitation, in terms of protocol, was that rather than build a streamlined, low-mass and portable version of our device (e.g.*,* as was done in [10]) we opted to use a high-powered, tethered ankle exoskeleton system to emulate and assess the effect of passive-elastic exoskeleton stiffness on the neuromechanics and energetics of walking across speeds. Our primary goal here was to employ a framework to rapidly and robustly test the specific effects of ankle exoskeleton stiffness independent of the mass and inertia of the device with high repeatability. This choice restricted our ability to definitively claim that the savings we measured with respect to the no assistance baseline condition (i.e.*,* k_exo_ = 0 Nm rad^− 1^ or zero-torque) would transfer to real savings with respect to normal walking in a portable version of the device using similar stiffness. For the tethered exoskeleton emulator we used here (Fig. 1), the increase in metabolic cost due to merely donning the system was on average ~ 19% (Supp. Table 1). We note, however, that our previous work indicates it is possible to build a passive-elastic system with negligible added mass cost [10], making the effect of the spring stiffness the primary variable of interest, and perhaps a more useful benchmark for extrapolating our results to expected performance on a portable analogue. Thus, our results suggest it should be possible to reduce metabolic cost of walking at both slow and fast speeds by ~ 5% using an unpowered, passive- elastic ankle exoskeleton with fixed rotational stiffness ~ 70–80 Nm rad^− 1^ (Fig. 2).

## Conclusions

In a move toward ‘real-world’ application, we examined whether the metabolic benefit of walking with unpowered elastic ankle exoskeletons extends across a range of walking speeds, and if so, whether the optimal exoskeleton stiffness was speed dependent. We showed that users were able to achieve a reduction in net metabolic power at slow (4.2% at 1.25 m s^− 1^) and fast (4.7% at 1.75 m s^− 1^) walking speeds but were not able to get a metabolic reduction at 1.5 m s^− 1^. These data suggest that the effect of elastic exoskeleton assistance varies with walking speed and is potentially limited at speeds close to the preferred (i.e.*,* nominally metabolically optimal) walking speed. In addition, contrary to our initial hypothesis, we found no appreciable increase in optimal exoskeleton stiffness with increasing walking speed. For both slow and fast walking speeds, optimal stiffness was between 50 and 100 Nm rad^− 1^. These findings help provide an initial ‘road-map’ for how ankle exoskeleton impedance should be adjusted (or not!) to match the changing mechanical demands of locomotion in ‘real-world’ environments. We suspect that studies designed to look ‘under the skin’ using computational modeling or ultrasound imaging and examine how exoskeleton stiffness influences biological muscle-tendon dynamics will yield further insight about how to optimally blend the impedance of an exoskeleton with that of a human joint.

## Supplementary information

**Additional file 1: Supplementary Figures and Tables**. **Supplementary Figure 1:** Ankle joint mechanics across stiffness and speed. **Supplementary Figure 2:** Muscle activity across stiffness and speed. **Supplementary Figure 3:** Knee joint mechanics across stiffness and speed. **Supplementary Figure 4:** Hip joint mechanics across stiffness and speed. **Supplementary Figure 5:** Net metabolic rate during exoskeleton training at 1.25 m s^-1^. **Supplementary Table 1**: Steady-state net metabolic rate for each subject, condition, and speed. **Supplementary Table 2**: Summary statistics for exoskeleton stiffness effect at 1.25 m s^-1^. **Supplementary Table 3**: Summary statistics for exoskeleton stiffness effect at 1.50 m s^-1^. **Supplementary Table 4**: Summary statistics for exoskeleton stiffness effect at 1.75 m s^-1^.

## Data Availability

Source data from this study in .mat and .txt format and an associated readme.txt for navigating it are available for download at: http://pwp.gatech.edu/hpl/archival-data-from-publications/

## Abbreviations

EMG: Electromyography
GRF: Ground reaction force
SOL: Soleus
MG: Medial Gastrocnemius
LG: Lateral Gastrocnemius
TA: Tibialis Anterior
BFL: Biceps Femoris Longhead
RF: Rectus Femoris
IRB: Institutional review board
