# Supplementary material for: Impact of elastic ankle exoskeleton stiffness on neuromechanics and energetics of human walking across multiple speeds
Source: J Neuroeng Rehabil. 2020 Jun 15;17:75. doi: 10.1186/s12984-020-00703-4 (PMC7294672; doi:10.1186/s12984-020-00703-4)
Supplement: Supplementary file 1 — Additional file 1: Supplementary Figures and Tables. Supplementary Figure 1: Ankle joint mechanics across stiffness and speed. Supplementary Figure 2: Muscle activity across stiffness and speed. Supplementary Figure 3: Knee joint mechanics across stiffness and speed. Supplementary Figure 4: Hip joint mechanics across stiffness and speed. Supplementary Figure 5: Net metabolic rate during exoskeleton training at 1.25 m s-1. Supplementary Table 1: Steady-state net metabolic rate for each subject, condition, and speed. Supplementary Table 2: Summary statistics for exoskeleton stiffness effect at 1.25 m s-1. Supplementary Table 3: Summary statistics for exoskeleton stiffness effect at 1.50 m s-1. Supplementary Table 4: Summary statistics for exoskeleton stiffness effect at 1.75 m s-1. [file 12984_2020_703_MOESM1_ESM.docx]

**Supplementary Figures and Tables:**

**Title**

Impact of elastic ankle exoskeleton stiffness on neuromechanics and energetics of human walking across multiple speeds

**Authors**

Richard W. Nuckols

Gregory S. Sawicki


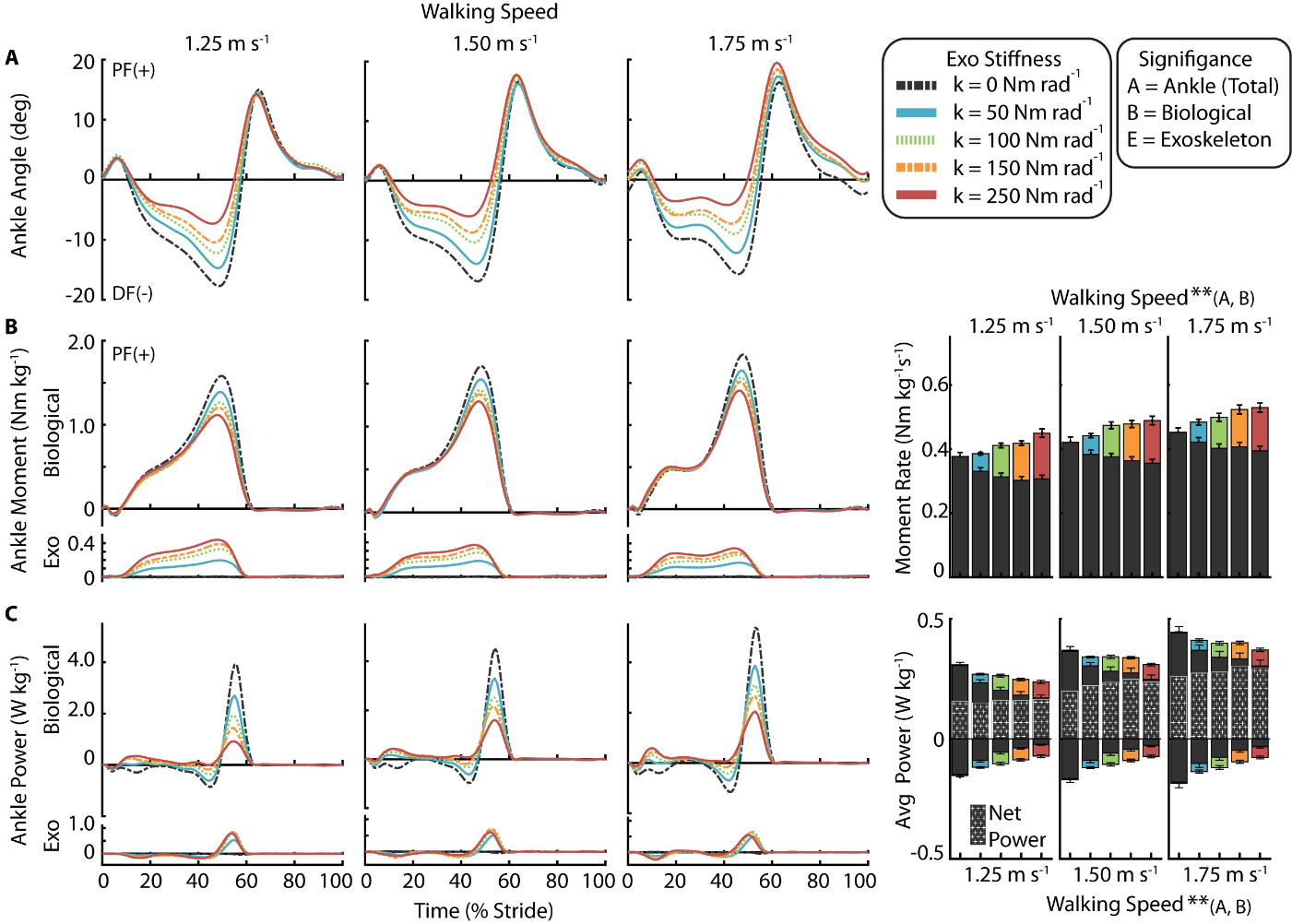


Supplementary Figure 1: Ankle joint mechanics across stiffness and speed. (A) Ankle joint angle became less dorsiflexed (*p* < 0.01) and more plantarflexed (*p* < 0.0001) with increased stiffness and speed. Time series of ankle joint plantarflexion (PF) and dorsiflexion (DF) angle for each stiffness and speed averaged across participants. (B) Exoskeleton peak torque decreased with increasing speed (*p* < 0.01) while total ankle and biological ankle moment increased with speed (*p* < 0.0001). Mass normalized biological ankle moment and exoskeleton torque for each stiffness and speed. Stacked bar charts represent the average biological (black-lower) and exoskeleton (colors-upper) contribution to total ankle moment for each speed over the stride. (C) Peak total ankle and biological ankle mechanical power increased with speed (*p* < 0.0001). Positive and net ankle mechanical power increased with increasing walking speed. Time series of 6-DOF biological ankle mechanical power and exoskeleton mechanical power for each stiffness and speed. Stacked charts represent biological and exoskeleton contribution to total ankle mechanical power and net total ankle mechanical power is the dashed insert. [main effect: speed ** *p* < 0.0001 B = Biological, E = Exo, A = Total Ankle]


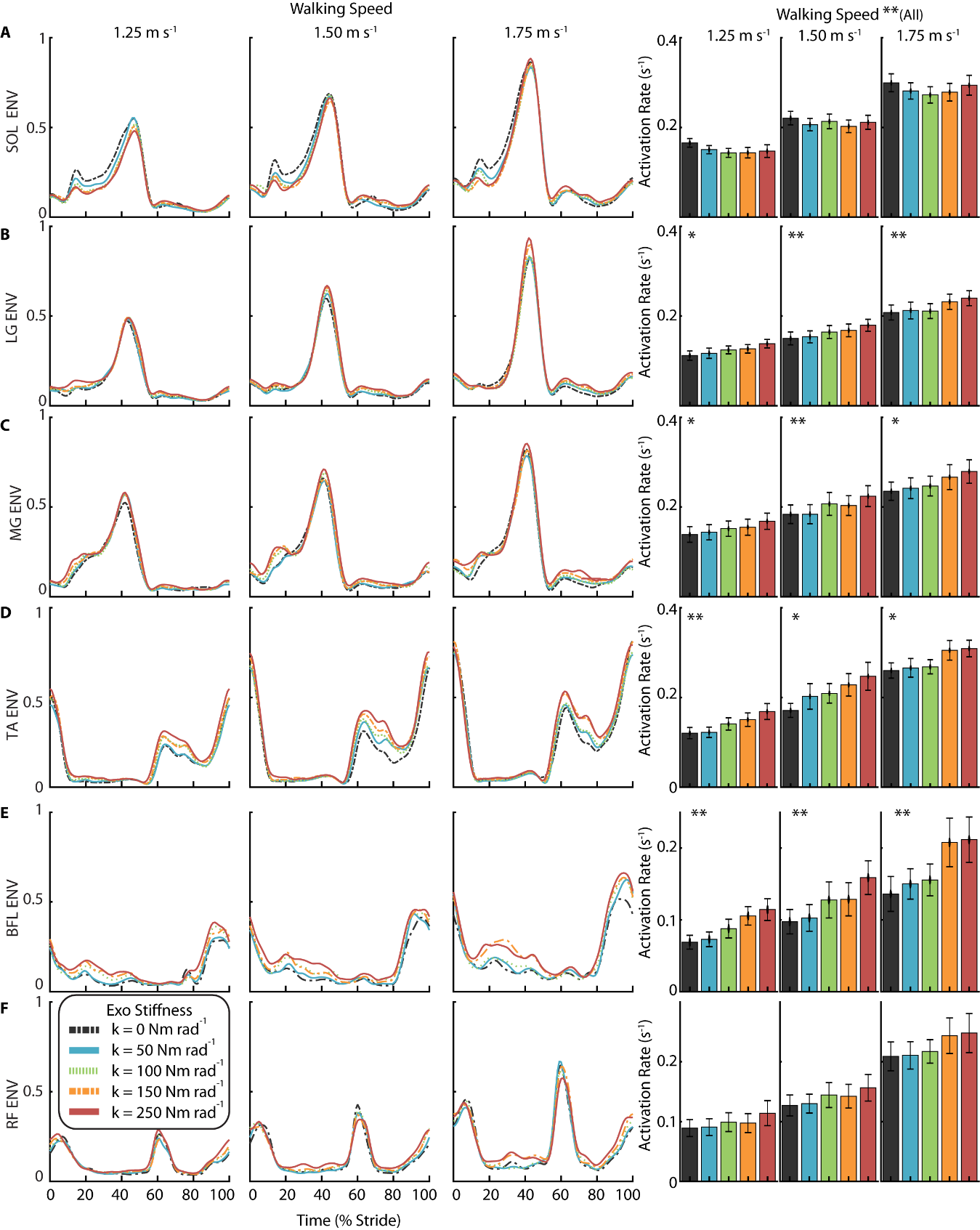


Supplementary Figure 2: Muscle activity across stiffness and speed. Time series of linear envelope electromyography recordings. Amplitude normalized to the peak value across stiffness and speed for each participant. Bar charts show average activation rate the stride. Amplitude normalized to the peak value across stiffness and speed for each participant. [main effect: stiffness ** *p* < 0.0001; * *p* < 0.05; speed ** *p* < 0.0001 for all muscles]


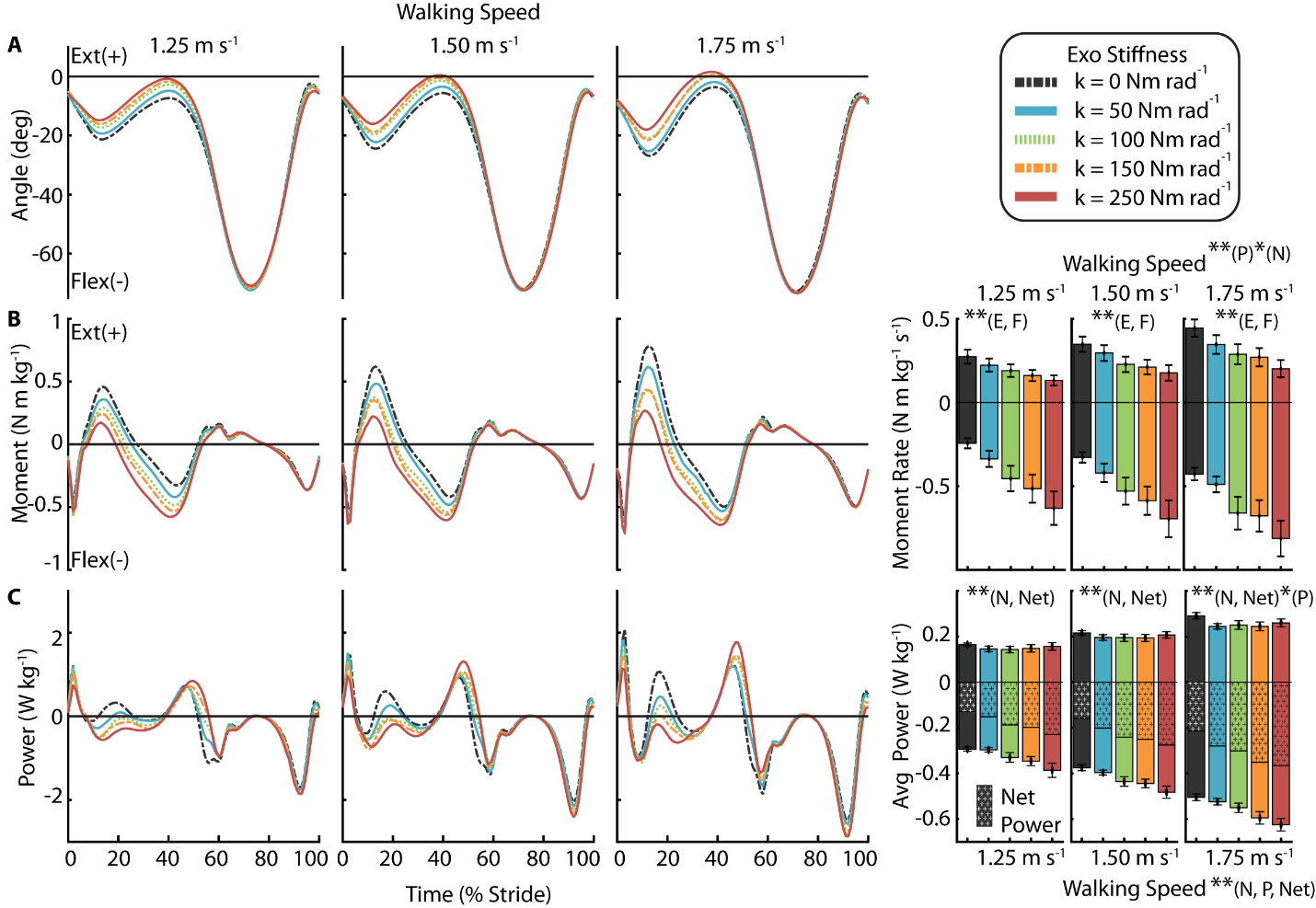


Supplementary Figure 3: Knee joint mechanics across stiffness and speed. (A) Time series of knee joint angle for each stiffness and speed averaged across participants. Knee angle became more extended with increased exoskeleton stiffness. (B) Mass normalized knee moment for each stiffness and speed. Bar charts represent the average extension (+) and flexion (-) moment over the stride. With increased stiffness, knee moment generally increased in flexion. Knee moment amplitude was larger at faster speed. (C) Time series of 6-DOF knee mechanical power for each stiffness and speed. Negative knee joint mechanical power increased with increasing stiffness and speed. [main effect: stiffness ** *p* < 0.0001, * *p* < 0.05; main effect: speed ** *p* < 0.0001, * *p* < 0.05; P = Positive, N = Negative]


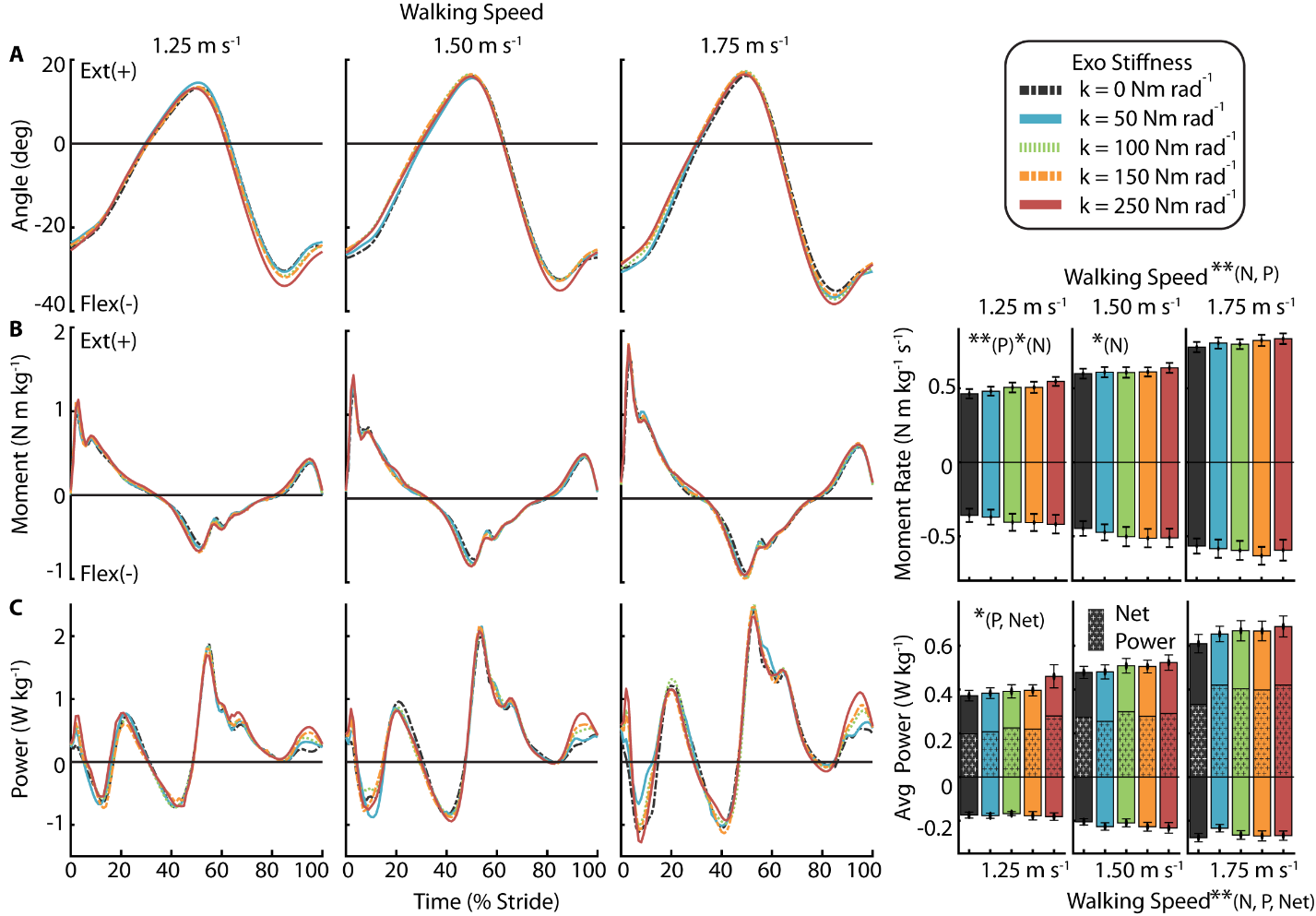


Supplementary Figure 4: Hip joint mechanics across stiffness and speed. (A) Time series of hip joint angle for each stiffness and speed averaged across participants. Hip angle was slightly more flexed prior to heel strike with higher exoskeleton stiffness. (B) Mass normalized moment for each stiffness and speed. Bar charts represent the average extension (+) and flexion (-) moment over the stride. Hip moment is slightly greater at faster speeds. (C) Time series of 6-DOF hip mechanical power for each stiffness and speed. Hip positive mechanical power increases with speed. [main effect: stiffness ** *p* < 0.0001, * *p* < 0.05; main effect: speed ** *p* < 0.0001, * *p* < 0.05; P = Positive, N = Negative]


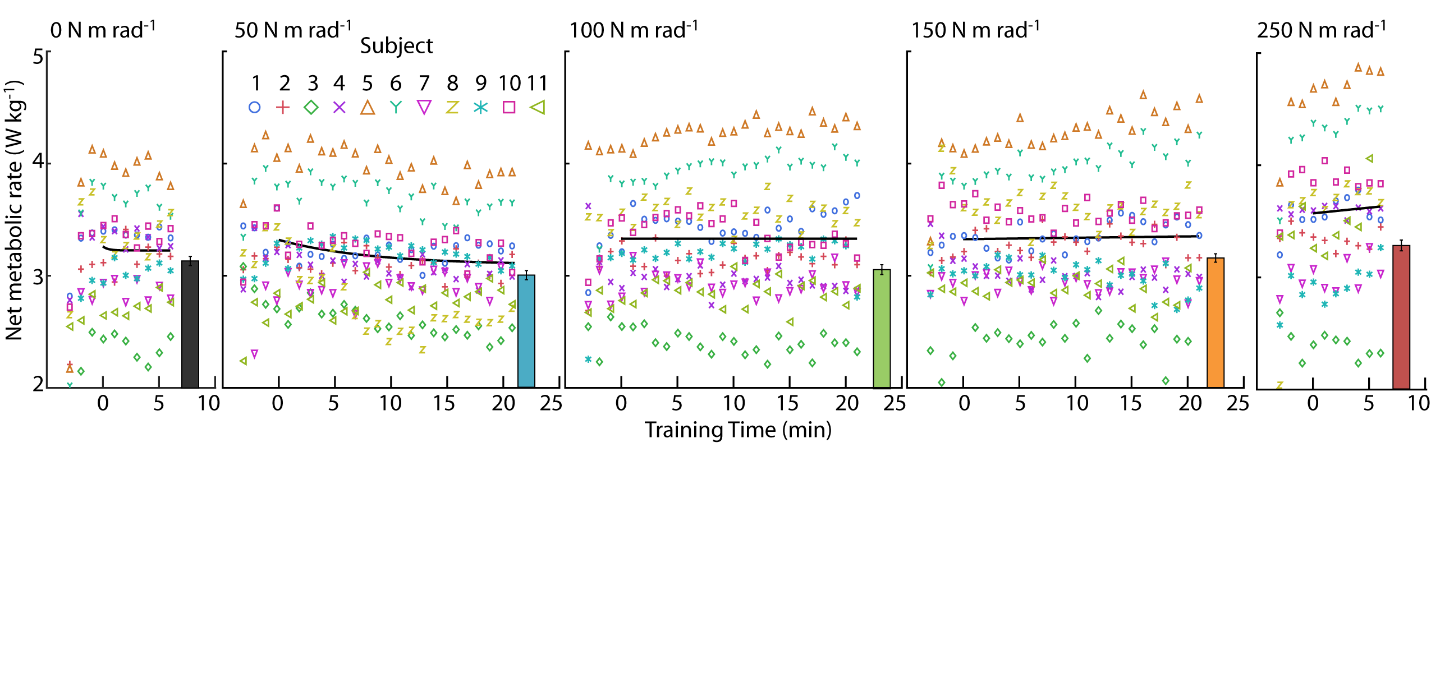


Supplementary Figure 5: Net metabolic rate during exoskeleton training at 1.25 m s^-1^. Each symbol represents the subject’s average metabolic rate data for each minute of training. At each stiffness an exponential curve was fit to the data. The only condition where subjects showed substantial improvement in net metabolic rate from training was the 50 N m rad^-1^ stiffness where we measured an improvement of 6.7% at the 25^th^ minute (n = 11, paired t-test, *p* = 0.017).

**Supplementary Table 1: Steady-state net metabolic rate for each subject, condition, and speed.**

|  |  | **Subject** | | | | | | | | | | |  |  |
| --- | --- | --- | --- | --- | --- | --- | --- | --- | --- | --- | --- | --- | --- | --- |
| **Walking Speed (m s^-1^)** | **Stiffness (Nm rad^-1^)** | **1** | **2** | **3** | **4** | **5** | **6** | **7** | **8** | **9** | **10** | **11** | **Mean** | **SEM** |
| **1.25** | **0** | 2.36 | 3.03 | 2.99 | 2.68 | 4.04 | 3.63 | 3.28 | 3.01 | 3.28 | 3.08 | 3.05 | **3.13** | 0.13 |
|  | **50** | **2.23** | **2.99** | 3.02 | **2.39** | **3.91** | **3.19** | **3.21** | 3.25 | **3.06** | **2.87** | **2.87** | **3.00** | 0.13 |
|  | **100** | **2.24** | **2.83** | **2.90** | **2.46** | **3.96** | **3.54** | **3.08** | 3.26 | **3.11** | **2.95** | 3.34 | **3.06** | 0.14 |
|  | **150** | 2.68 | 3.22 | **2.81** | **2.52** | **3.98** | **3.59** | **3.19** | 3.10 | **3.25** | **3.06** | 3.34 | **3.16** | 0.12 |
|  | **250** | 2.60 | 3.10 | **2.88** | **2.63** | 4.25 | 4.07 | **3.13** | 3.55 | 3.46 | 3.25 | 3.14 | **3.28** | 0.16 |
|  | **NE** | **1.95** | **2.71** | **2.22** | **2.25** | **3.29** | **2.89** | **2.87** | **2.62** | **2.88** | **2.63** | **2.83** | **2.65** | 0.11 |
| **1.5** | **0** | 3.14 | 3.99 | 3.32 | 3.42 | 5.23 | 4.58 | 4.05 | 3.92 | 4.75 | 3.99 | 3.68 | **4.01** | 0.19 |
|  | **50** | **3.11** | 4.33 | 3.51 | **3.37** | 5.28 | **4.40** | 4.08 | 4.07 | **4.41** | **3.96** | 3.82 | **4.03** | 0.18 |
|  | **100** | **3.08** | 4.03 | 3.80 | **3.38** | 5.20 | **4.57** | 4.19 | 4.22 | **4.05** | **3.78** | 4.26 | **4.05** | 0.17 |
|  | **150** | 3.18 | 4.39 | 3.79 | 3.45 | 5.52 | 5.17 | **4.02** | 4.08 | **4.53** | **3.90** | 4.32 | **4.21** | 0.21 |
|  | **250** | **3.08** | 4.52 | **3.30** | 3.62 | 5.62 | 5.14 | 4.29 | 4.45 | **4.58** | 4.13 | 4.50 | **4.29** | 0.23 |
|  | **NE** | **2.37** | **3.77** | **2.74** | **3.16** | **4.35** | **3.76** | **2.82** | **3.44** | **3.70** | **3.37** | **3.39** | **3.35** | 0.17 |
| **1.75** | **0** | 4.06 | 5.38 | 5.31 | 5.08 | 7.32 | 5.97 | 6.24 | 5.63 | 6.67 | 5.23 | 5.26 | **5.65** | 0.26 |
|  | **50** | 4.10 | **5.08** | **4.91** | **4.95** | **6.75** | **5.46** | 6.28 | **5.39** | **5.92** | **5.05** | 5.34 | **5.38** | 0.22 |
|  | **100** | 4.30 | 5.42 | **4.84** | **4.76** | **6.62** | 6.02 | **6.23** | **5.33** | **6.04** | 5.29 | 5.39 | **5.48** | 0.21 |
|  | **150** | 4.14 | 5.64 | **4.82** | **4.86** | **7.02** | 6.55 | 6.63 | **5.47** | **6.56** | **5.19** | 5.56 | **5.68** | 0.28 |
|  | **250** | 4.40 | 5.45 | **5.10** | **4.94** | **7.06** | 6.84 | 7.35 | 5.66 | **6.59** | 5.36 | 5.69 | **5.86** | 0.29 |
|  | **NE** | **3.50** | **4.91** | **4.05** | **4.59** | **5.61** | **5.24** | **5.06** | **4.85** | **5.18** | **4.82** | **4.51** | **4.76** | 0.18 |

Highlighted cells represent conditions where the subject’s net metabolic rate was less than the no assistance (*i.e.,* k_exo_ = 0 N-m rad ^-1^) condition. Due to the significant added-mass cost to wear the tethered ankle exoskeleton hardware, users’ net metabolic rate in the no exoskeleton (NE) condition *(i.e.*, normal walking without the device) was less than any exoskeleton condition across all speeds. Fewer conditions were metabolically beneficial in the 1.5 m s^-1^ walking speed

**Supplementary Table 2: Summary statistics for exoskeleton stiffness effect at 1.25 m s^-1^ (mean ± s.e.m**).

| Walking Speed: 1.25 m s^-1^ | | | | | | | | |
| --- | --- | --- | --- | --- | --- | --- | --- | --- |
|  |  |  | Effect | Exoskeleton Stiffness (Nm rad^-1^) | | | | |
|  |  |  | k_exo_ | 0 | 50 | 100 | 150 | 250 |
| Ankle Moment  Rate  (Nm  kg^-1^ s^-1^) | Stride | Total | *p* < 0.0001 | 0.37 ± 0.01 | 0.38 ± 0.01 | 0.41 ± 0.01 | 0.41 ± 0.02 | 0.44 ± 0.02 |
|  |  | Bio | *p* < 0.0001 | 0.38 ± 0.01 | 0.33 ± 0.01 | 0.31 ± 0.01 | 0.3 ± 0.01 | 0.31 ± 0.01 |
|  |  | Exo | *p* < 0.0001 | 0 ± 0 | 0.06 ± 0 | 0.1 ± 0.01 | 0.12 ± 0.01 | 0.14 ± 0.01 |
|  | Early Stance | Total | *p* < 0.0001 | 0.93 ± 0.07 | 1.02 ± 0.08 | 1.15 ± 0.08 | 1.21 ± 0.09 | 1.41 ± 0.11 |
|  |  | Bio | *p* = 0.0285 | 0.95 ± 0.07 | 0.86 ± 0.07 | 0.85 ± 0.07 | 0.84 ± 0.07 | 0.93 ± 0.07 |
|  |  | Exo | *p* < 0.0001 | -0.01 ± 0 | 0.17 ± 0.02 | 0.31 ± 0.03 | 0.37 ± 0.03 | 0.49 ± 0.06 |
|  | Peak | Total | - | 1.59 ± 0.04 | 1.59 ± 0.03 | 1.6 ± 0.04 | 1.6 ± 0.04 | 1.57 ± 0.04 |
|  |  | Bio | *p* < 0.0001 | 1.6 ± 0.04 | 1.41 ± 0.03 | 1.29 ± 0.04 | 1.23 ± 0.04 | 1.14 ± 0.04 |
|  |  | Exo | *p* < 0.0001 | 0.01 ± 0 | 0.2 ± 0.01 | 0.34 ± 0.03 | 0.4 ± 0.02 | 0.47 ± 0.03 |
| Positive Power  (W kg^-1^) | Stride | Total | *p* < 0.0001 | 0.3 ± 0.01 | 0.26 ± 0.01 | 0.25 ± 0.01 | 0.23 ± 0.02 | 0.21 ± 0.01 |
|  |  | Bio | *p* < 0.0001 | 0.31 ± 0.01 | 0.23 ± 0.01 | 0.2 ± 0.01 | 0.18 ± 0.02 | 0.17 ± 0.01 |
|  |  | Exo | - | 0 ± 0 | 0.04 ± 0 | 0.06 ± 0.01 | 0.07 ± 0.01 | 0.07 ± 0.01 |
| Muscle Activation  Rate  (s^-1^) | Stride | SOL | - | 0.17 ± 0.01 | 0.15 ± 0.01 | 0.14 ± 0.01 | 0.14 ± 0.01 | 0.15 ± 0.01 |
|  |  | SOL + TA | - | 0.29 ± 0.02 | 0.27 ± 0.02 | 0.28 ± 0.02 | 0.29 ± 0.02 | 0.32 ± 0.03 |
|  |  | TA | *p* < 0.0001 | 0.12 ± 0.01 | 0.12 ± 0.01 | 0.14 ± 0.01 | 0.15 ± 0.02 | 0.17 ± 0.02 |
|  |  | MG | *p* = 0.001 | 0.14 ± 0.02 | 0.14 ± 0.02 | 0.15 ± 0.02 | 0.16 ± 0.02 | 0.17 ± 0.02 |
|  |  | LG | *p* = 0.0003 | 0.11 ± 0.01 | 0.12 ± 0.01 | 0.12 ± 0.01 | 0.13 ± 0.01 | 0.14 ± 0.01 |
|  |  | BFL | *p* < 0.0001 | 0.07 ± 0.01 | 0.07 ± 0.01 | 0.09 ± 0.01 | 0.11 ± 0.01 | 0.11 ± 0.02 |
|  |  | RF | - | 0.09 ± 0.01 | 0.09 ± 0.01 | 0.1 ± 0.02 | 0.1 ± 0.02 | 0.11 ± 0.02 |
| Muscle Activation | Stride | SOL | *p* = 0.0004 | 0.18 ± 0.01 | 0.16 ± 0.01 | 0.15 ± 0.01 | 0.15 ± 0.01 | 0.15 ± 0.01 |
|  |  | SOL + TA | *p* = 0.0412 | 0.31 ± 0.02 | 0.29 ± 0.02 | 0.3 ± 0.02 | 0.31 ± 0.02 | 0.32 ± 0.02 |
|  |  | TA | *p* < 0.0001 | 0.13 ± 0.01 | 0.13 ± 0.01 | 0.15 ± 0.01 | 0.16 ± 0.02 | 0.17 ± 0.02 |
|  |  | MG | *p* = 0.0044 | 0.15 ± 0.02 | 0.16 ± 0.02 | 0.16 ± 0.02 | 0.16 ± 0.02 | 0.17 ± 0.02 |
|  |  | LG | *p* = 0.0015 | 0.12 ± 0.01 | 0.13 ± 0.01 | 0.13 ± 0.01 | 0.13 ± 0.01 | 0.14 ± 0.01 |
|  |  | BFL | *p* < 0.0001 | 0.1 ± 0.01 | 0.1 ± 0.01 | 0.12 ± 0.01 | 0.13 ± 0.01 | 0.14 ± 0.01 |
|  |  | RF | - | 0.1 ± 0.02 | 0.1 ± 0.02 | 0.11 ± 0.02 | 0.1 ± 0.02 | 0.12 ± 0.02 |
| Range of Motion  (deg) | Peak | Plantar  flexion | - | 15.17 ± 1.44 | 14.71 ± 1.22 | 14.85 ± 1.45 | 14.46 ± 1.49 | 14.53 ± 1.26 |
|  |  | Dorsi-flexion | *p* < 0.0001 | -17.88 ± 1.24 | -15.04 ± 1.26 | -12.55 ± 1.38 | -10.9 ± 1.4 | -8.12 ± 1.39 |
| Spatio-Temporal  (sec) | Time | Stride | *p* = 0.0063 | 1.09 ± 0.02 | 1.08 ± 0.02 | 1.07 ± 0.03 | 1.07 ± 0.03 | 1.04 ± 0.03 |
|  |  | Stance | *p* = 0.0002 | 0.7 ± 0.01 | 0.7 ± 0.01 | 0.69 ± 0.02 | 0.68 ± 0.02 | 0.67 ± 0.02 |
|  |  | Duty % | *p* = 0.0312 | 64.5 ± 0.25 | 64.5 ± 0.28 | 64.3 ± 0.19 | 64 ± 0.23 | 63.8 ± 0.26 |

**Supplementary Table 3: Summary statistics for exoskeleton stiffness effect at 1.50 m s^-1^ (mean ± s.e.m**).

| Walking Speed: 1.50 m s^-1^ | | | | | | | | |
| --- | --- | --- | --- | --- | --- | --- | --- | --- |
|  |  |  | Effect | Exoskeleton Stiffness (Nm rad^-1^) | | | | |
|  |  |  | k_exo_ | 0 | 50 | 100 | 150 | 250 |
| Ankle Moment  Rate  (Nm  kg^-1^ s^-1^) | Stride | Total | p < 0.0001 | 0.41 ± 0.02 | 0.44 ± 0.01 | 0.47 ± 0.01 | 0.47 ± 0.02 | 0.48 ± 0.02 |
|  |  | Bio | p < 0.0001 | 0.42 ± 0.02 | 0.38 ± 0.01 | 0.38 ± 0.01 | 0.36 ± 0.01 | 0.36 ± 0.01 |
|  |  | Exo | p < 0.0001 | 0 ± 0 | 0.06 ± 0.01 | 0.1 ± 0.01 | 0.11 ± 0.01 | 0.13 ± 0.01 |
|  | Early Stance | Total | p < 0.0001 | 1.08 ± 0.1 | 1.22 ± 0.1 | 1.42 ± 0.12 | 1.48 ± 0.11 | 1.59 ± 0.13 |
|  |  | Bio | - | 1.11 ± 0.11 | 1.03 ± 0.09 | 1.08 ± 0.09 | 1.08 ± 0.08 | 1.09 ± 0.09 |
|  |  | Exo | p < 0.0001 | -0.01 ± 0 | 0.21 ± 0.02 | 0.36 ± 0.05 | 0.42 ± 0.05 | 0.52 ± 0.06 |
|  | Peak | Total | - | 1.72 ± 0.05 | 1.75 ± 0.04 | 1.74 ± 0.05 | 1.73 ± 0.05 | 1.69 ± 0.05 |
|  |  | Bio | p < 0.0001 | 1.73 ± 0.05 | 1.58 ± 0.04 | 1.47 ± 0.05 | 1.41 ± 0.05 | 1.34 ± 0.06 |
|  |  | Exo | p < 0.0001 | 0.01 ± 0 | 0.2 ± 0.01 | 0.31 ± 0.03 | 0.37 ± 0.02 | 0.44 ± 0.03 |
| Positive Power  (W kg^-1^) | Stride | Total | p < 0.0001 | 0.36 ± 0.02 | 0.33 ± 0.01 | 0.33 ± 0.02 | 0.32 ± 0.02 | 0.29 ± 0.02 |
|  |  | Bio | p < 0.0001 | 0.36 ± 0.02 | 0.3 ± 0.01 | 0.28 ± 0.02 | 0.27 ± 0.02 | 0.25 ± 0.02 |
|  |  | Exo | p < 0.0001 | 0 ± 0 | 0.04 ± 0 | 0.06 ± 0.01 | 0.06 ± 0 | 0.06 ± 0.01 |
| Muscle Activation  Rate  (s^-1^) | Stride | SOL | - | 0.22 ± 0.02 | 0.21 ± 0.01 | 0.21 ± 0.02 | 0.2 ± 0.01 | 0.21 ± 0.02 |
|  |  | SOL + TA | - | 0.39 ± 0.03 | 0.41 ± 0.03 | 0.42 ± 0.04 | 0.43 ± 0.04 | 0.46 ± 0.04 |
|  |  | TA | p = 0.0032 | 0.17 ± 0.02 | 0.2 ± 0.03 | 0.21 ± 0.02 | 0.23 ± 0.03 | 0.25 ± 0.03 |
|  |  | MG | p < 0.0001 | 0.18 ± 0.02 | 0.18 ± 0.02 | 0.21 ± 0.03 | 0.2 ± 0.02 | 0.22 ± 0.02 |
|  |  | LG | p < 0.0001 | 0.15 ± 0.01 | 0.15 ± 0.01 | 0.16 ± 0.01 | 0.17 ± 0.01 | 0.18 ± 0.01 |
|  |  | BFL | p < 0.0001 | 0.13 ± 0.02 | 0.14 ± 0.01 | 0.16 ± 0.02 | 0.17 ± 0.02 | 0.19 ± 0.02 |
|  |  | RF | - | 0.13 ± 0.02 | 0.13 ± 0.02 | 0.14 ± 0.02 | 0.14 ± 0.02 | 0.16 ± 0.02 |
| Muscle Activation | Stride | SOL | - | 0.22 ± 0.01 | 0.2 ± 0.01 | 0.21 ± 0.01 | 0.2 ± 0.01 | 0.2 ± 0.01 |
|  |  | SOL + TA | - | 0.39 ± 0.02 | 0.4 ± 0.03 | 0.41 ± 0.03 | 0.42 ± 0.03 | 0.44 ± 0.03 |
|  |  | TA | *p* = 0.0036 | 0.17 ± 0.01 | 0.2 ± 0.03 | 0.2 ± 0.02 | 0.22 ± 0.02 | 0.24 ± 0.02 |
|  |  | MG | *p* < 0.0001 | 0.18 ± 0.02 | 0.18 ± 0.02 | 0.2 ± 0.02 | 0.2 ± 0.02 | 0.22 ± 0.02 |
|  |  | LG | *p* < 0.0001 | 0.15 ± 0.01 | 0.15 ± 0.01 | 0.16 ± 0.01 | 0.16 ± 0.01 | 0.17 ± 0.01 |
|  |  | BFL | *p* < 0.0001 | 0.13 ± 0.02 | 0.14 ± 0.02 | 0.16 ± 0.02 | 0.16 ± 0.02 | 0.19 ± 0.02 |
|  |  | RF | - | 0.13 ± 0.02 | 0.13 ± 0.02 | 0.14 ± 0.02 | 0.14 ± 0.02 | 0.15 ± 0.02 |
| Range of Motion  (deg) | Peak | Plantar  flexion | - | 16.84 ± 1.36 | 16.57 ± 1.31 | 17.36 ± 1.25 | 17.86 ± 1.34 | 18.04 ± 1.46 |
|  |  | Dorsi-flexion | p < 0.0001 | -17.38 ± 0.67 | -14.59 ± 0.86 | -11.91 ± 1.46 | -10.17 ± 1.42 | -7.88 ± 1.73 |
| Spatio-Temporal  (sec) | Time | Stride | p = 0.001 | 1 ± 0.02 | 1 ± 0.02 | 0.98 ± 0.02 | 0.99 ± 0.02 | 0.98 ± 0.02 |
|  |  | Stance | p < 0.0001 | 0.63 ± 0.01 | 0.63 ± 0.01 | 0.61 ± 0.01 | 0.61 ± 0.01 | 0.61 ± 0.02 |
|  |  | Duty % | p = 0.0008 | 63.18 ± 0.23 | 62.91 ± 0.28 | 62.45 ± 0.25 | 62.36 ± 0.2 | 62.55 ± 0.28 |

**Supplementary Table 4: Summary statistics for exoskeleton stiffness effect at 1.75 m s^-1^ (mean ± s.e.m**).

| Walking Speed: 1.75 m s^-1^ | | | | | | | | |
| --- | --- | --- | --- | --- | --- | --- | --- | --- |
|  |  |  | Effect | Exoskeleton Stiffness (Nm rad^-1^) | | | | |
|  |  |  | k_exo_ | 0 | 50 | 100 | 150 | 250 |
| Ankle Moment  Rate  (Nm  kg^-1^ s^-1^) | Stride | Total | p < 0.0001 | 0.44 ± 0.01 | 0.48 ± 0.02 | 0.49 ± 0.02 | 0.52 ± 0.02 | 0.52 ± 0.02 |
|  |  | Bio | p < 0.0001 | 0.45 ± 0.01 | 0.42 ± 0.01 | 0.4 ± 0.01 | 0.41 ± 0.01 | 0.39 ± 0.01 |
|  |  | Exo | p < 0.0001 | 0 ± 0 | 0.06 ± 0.01 | 0.1 ± 0.01 | 0.12 ± 0.01 | 0.13 ± 0.01 |
|  | Early Stance | Total | p < 0.0001 | 1.12 ± 0.09 | 1.38 ± 0.14 | 1.49 ± 0.11 | 1.64 ± 0.15 | 1.77 ± 0.14 |
|  |  | Bio | - | 1.14 ± 0.09 | 1.17 ± 0.11 | 1.14 ± 0.08 | 1.19 ± 0.1 | 1.23 ± 0.1 |
|  |  | Exo | p < 0.0001 | -0.01 ± 0 | 0.23 ± 0.04 | 0.37 ± 0.05 | 0.47 ± 0.07 | 0.56 ± 0.07 |
|  | Peak | Total | - | 1.85 ± 0.06 | 1.84 ± 0.06 | 1.84 ± 0.06 | 1.82 ± 0.07 | 1.78 ± 0.07 |
|  |  | Bio | p < 0.0001 | 1.86 ± 0.06 | 1.69 ± 0.06 | 1.6 ± 0.07 | 1.55 ± 0.08 | 1.45 ± 0.08 |
|  |  | Exo | p < 0.0001 | 0.01 ± 0 | 0.19 ± 0.02 | 0.29 ± 0.03 | 0.36 ± 0.03 | 0.42 ± 0.03 |
| Positive Power  (W kg^-1^) | Stride | Total | p < 0.0001 | 0.44 ± 0.03 | 0.4 ± 0.02 | 0.39 ± 0.02 | 0.38 ± 0.02 | 0.35 ± 0.02 |
|  |  | Bio | p < 0.0001 | 0.44 ± 0.03 | 0.37 ± 0.02 | 0.34 ± 0.02 | 0.33 ± 0.02 | 0.3 ± 0.02 |
|  |  | Exo | p < 0.0001 | 0 ± 0 | 0.04 ± 0.01 | 0.06 ± 0.01 | 0.07 ± 0.01 | 0.07 ± 0.01 |
| Muscle Activation  Rate  (s^-1^) | Stride | SOL | - | 0.3 ± 0.02 | 0.28 ± 0.02 | 0.27 ± 0.02 | 0.28 ± 0.02 | 0.29 ± 0.02 |
|  |  | SOL + TA | - | 0.56 ± 0.04 | 0.55 ± 0.03 | 0.54 ± 0.03 | 0.58 ± 0.04 | 0.6 ± 0.04 |
|  |  | TA | p < 0.0001 | 0.26 ± 0.02 | 0.27 ± 0.02 | 0.27 ± 0.02 | 0.31 ± 0.02 | 0.31 ± 0.02 |
|  |  | MG | p = 0.0006 | 0.24 ± 0.02 | 0.24 ± 0.02 | 0.25 ± 0.02 | 0.27 ± 0.03 | 0.28 ± 0.03 |
|  |  | LG | p < 0.0001 | 0.21 ± 0.02 | 0.21 ± 0.02 | 0.21 ± 0.02 | 0.23 ± 0.02 | 0.24 ± 0.02 |
|  |  | BFL | p < 0.0001 | 0.2 ± 0.02 | 0.22 ± 0.02 | 0.23 ± 0.03 | 0.28 ± 0.04 | 0.29 ± 0.04 |
|  |  | RF | - | 0.21 ± 0.02 | 0.21 ± 0.02 | 0.22 ± 0.02 | 0.24 ± 0.03 | 0.25 ± 0.03 |
| Muscle Activation | Stride | SOL | - | 0.27 ± 0.01 | 0.26 ± 0.01 | 0.25 ± 0.01 | 0.25 ± 0.01 | 0.26 ± 0.01 |
|  |  | SOL + TA | - | 0.51 ± 0.03 | 0.5 ± 0.02 | 0.49 ± 0.02 | 0.52 ± 0.02 | 0.54 ± 0.02 |
|  |  | TA | *p* = 0.0398 | 0.24 ± 0.01 | 0.24 ± 0.02 | 0.25 ± 0.01 | 0.27 ± 0.01 | 0.28 ± 0.01 |
|  |  | MG | *p* = 0.0019 | 0.22 ± 0.02 | 0.22 ± 0.02 | 0.23 ± 0.02 | 0.24 ± 0.02 | 0.25 ± 0.02 |
|  |  | LG | *p* = 0.0005 | 0.19 ± 0.01 | 0.19 ± 0.02 | 0.19 ± 0.01 | 0.21 ± 0.01 | 0.22 ± 0.01 |
|  |  | BFL | *p* < 0.0001 | 0.18 ± 0.02 | 0.2 ± 0.02 | 0.21 ± 0.02 | 0.25 ± 0.03 | 0.26 ± 0.03 |
|  |  | RF | - | 0.19 ± 0.02 | 0.19 ± 0.02 | 0.2 ± 0.02 | 0.22 ± 0.03 | 0.22 ± 0.03 |
| Range of Motion  (deg) | Peak | Plantar  flexion | p = 0.0116 | 16.51 ± 1.41 | 17.61 ± 1.39 | 18.37 ± 1.37 | 18.61 ± 1.15 | 19.65 ± 1.21 |
|  |  | Dorsi-flexion | p < 0.0001 | -16.56 ± 1.01 | -13.72 ± 1.1 | -10.44 ± 1.39 | -9.94 ± 1.44 | -6.61 ± 1.55 |
| Spatio-Temporal  (sec) | Time | Stride | p = 0.0115 | 0.93 ± 0.02 | 0.92 ± 0.02 | 0.92 ± 0.02 | 0.91 ± 0.02 | 0.91 ± 0.02 |
|  |  | Stance | p = 0.0044 | 0.58 ± 0.01 | 0.57 ± 0.01 | 0.57 ± 0.01 | 0.56 ± 0.01 | 0.56 ± 0.01 |
|  |  | Duty % | - | 62.27 ± 0.24 | 62 ± 0.33 | 61.64 ± 0.28 | 61.64 ± 0.34 | 61.45 ± 0.41 |
